# Selenophene-containing heterotriacenes by a C–Se coupling/cyclization reaction

**DOI:** 10.3762/bjoc.15.138

**Published:** 2019-06-24

**Authors:** Pierre-Olivier Schwartz, Sebastian Förtsch, Astrid Vogt, Elena Mena-Osteritz, Peter Bäuerle

**Affiliations:** 1 Institute of Organic Chemistry II and Advanced Materials, University of Ulm, Albert-Einstein-Allee 11, 89081 Ulm, Germany; 2 Alsachim, 160 Rue Tobias Stimmer, 67400 Illkirch-Graffenstaden, France; 3 DuPont, August-Wolff-Straße 13, 29699 Bomlitz, Germany

**Keywords:** conducting polymer, C–S coupling, C–Se coupling, heteroacene, selenophene

## Abstract

A new novel family of tricyclic sulfur and/or selenium-containing heterotriacenes **2**–**4** with an increasing number of selenium (Se) atoms is presented. The heterotriacene derivatives were synthesized in multistep synthetic routes and the crucial cyclization steps to the stable and soluble fused systems were achieved by copper-catalyzed C–S and C–Se coupling/cyclization reactions. Structures and packing motifs in the solid state were elucidated by single crystal X-ray analysis and XRD powder measurements. Comparison of the optoelectronic properties provides interesting structure–property relationships and gives valuable insights into the role of heteroatoms within the series of the heterotriacenes. Electrooxidative polymerization led to the corresponding poly(heterotriacene)s **P2–P4**.

## Introduction

In recent years, great interest has been devoted to the development of new π-conjugated polycyclic molecules, in particular to polycyclic aromatic hydrocarbons (PAH) such as acenes [1], phenacenes [2], or nanographenes [3]. Corresponding heteroacenes incorporating heteroatoms such as nitrogen or sulfur represent encouraging alternatives to PAHs providing manageable electronic properties and increased chemical stability [4–5]. In this respect, series of heteroacenes consisting of fused five-membered heterocycles such as thienoacenes [6–7] or *S*,*N*-heteroacenes [8] were investigated and successfully used as building blocks for high-performance organic electronic materials and devices [9–16]. Among the different heteroatoms that can be introduced into heteroacenes and in contrast to corresponding thiophene-based systems, selenium (Se) has only sparingly been used most probably because of the high price of selenophene itself, the limited number of commercially available derivatives, and the less explored chemistry. Nevertheless, the implementation of selenophenes as heteroanalogues of thiophene-based materials is highly attractive, because molecules containing selenophene fragments instead of thiophene showed promising optical and electrochemical properties [17–19] and improved charge transport characteristics [20]. With respect to fused selenoloacenes, only the shortest parent system consisting of two fused heterocycles, mixed thieno[3,2-*b*]selenophene [21–22] and selenolo[3,2-*b*]selenophene [23], were described and represent analogues to the well-known thieno[3,2-*b*]thiophene [24]. Three fused selenophenes only were implemented in larger heteroacenes and analyzed towards their optical properties [25] whereupon the unsubstituted parent system, diselenolo[3,2-*b*:2’,3’-*d*]selenophene (DSS), is still unknown. Cheng et al. published a synthesis of various heterotriacenes including two selenophenes bridged with other elements such as silicon, germanium, nitrogen, and carbon [26]. Very recently, Wang et al. released selenophene-based heteroacenes via trimethylsilyl (TMS)-substituted selenolotriacenes, which served as intermediate building blocks [27].

**Figure 1:**
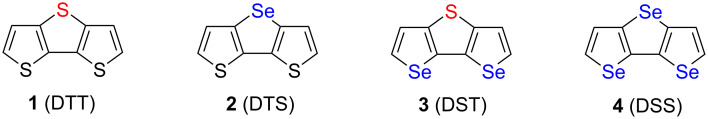
Heterotriacenes DTT **1**, DTS **2**, DST **3**, and DSS **4** with varying number of selenium atoms and fused selenophene rings.

In continuation of our work on heteroacenes, we now report synthesis and characterization of fused tricyclic selenium or selenophene-containing heteroacenes **2**–**4**, which represent the so far unknown unsubstituted parent systems of the selenolotriacenes synthesized by Wang et al. [27] and are analogues of the well-known dithieno[3,2-*b*:2’,3’-*d*]thiophene (**1**, DTT) [24]. These triacenes **2**–**4** contain an increasing number of selenium atoms and for their synthesis not only selenophene was used as starting material, but also ring fusion to selenophene was achieved by Cu-catalyzed C–Se cross-coupling reaction [28]. The detailed geometric structure and the packing behaviour in the solid state of triacenes **2**–**4** have been elucidated by single crystal X-ray structure analysis and X-ray diffraction on powders. Furthermore, the systematically varied structures of triacenes **1**–**4** allow for investigation of the influence of the number and position of selenium atoms or selenophene rings on the physical and electronic properties in fused systems (Figure 1).

## Results and Discussion

**Scheme 1:**
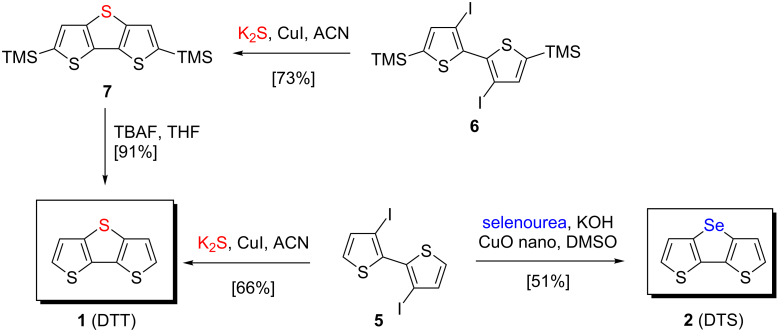
Synthesis of heterotriacenes DTT **1** and DTS **2** via copper-catalyzed cross-coupling reactions.

**Syntheses**. Several routes for the synthesis of dithienothiophene **1**, which is mostly built up by oxidative dehydrocoupling of 3,3’-dithienyl sulfide or ring-closure reactions of brominated thiophenes with ethyl mercaptoacetate, are described in literature [24]. For comparability to the selenophene-containing triacenes **2**–**4**, we reinvestigated the synthesis of DTT **1** by using a Cu-catalyzed C–S cross-coupling reaction with potassium sulfide (K_2_S) as sulfur source [29]. The best results for this C–S ring-closure reaction were achieved by reacting 3,3’-diiodo-2,2’-bithiophene (**5**) [30] with the system K_2_S and copper iodide (CuI) as catalyst in acetonitrile at 140 °C in a Schlenck tube to give DTT **1** in 66% yield. In the same way, trimethylsilyl (TMS)-protected diiodobithiophene **6** [31] gave 2,6-bis(trimethylsilyl)dithienothiophene **7** [32] in 73% yield, which was subsequently deprotected by tetrabutylammonium fluoride (TBAF) to form target DTT **1** in 91% yield (Scheme 1).

Triacene dithieno[3,2-*b*:2’,3’-*d*]selenophene (DTS, **2**) was successfully prepared as well from diiodinated bithiophene **5** in 51% yield after purification in a C–Se cross-coupling/cyclization reaction with selenourea as selenium source, copper oxide nanoparticles as catalyst, and potassium hydroxide as base in DMSO (Scheme 1). This method has been previously used for the synthesis of symmetrical diaryl selenides from aryl halides [28]. Attempts to use the corresponding 3,3’-dibromo-2,2’-bithiophene as starting material for the synthesis of either DTS **2** with the same reagents as aforementioned or DTT **1** with thiourea or thioacetate in a Pd-catalyzed reaction [33] led in both cases to substantially lower yields.

For the synthesis of selenolotriacenes (DST) **3** and (DSS) **4** we followed the same strategies and applied the above described Cu-catalyzed C–S and C–Se cross-coupling/cyclization reactions, respectively. In both cases, the synthesis started from TMS-protected diiodinated 2,2’-biselenophene **11**, which was prepared from 2-iodo-5-(trimethylsilyl)selenophene (**10**) in 59% yield by lithiation with LDA, halogen-dance reaction [34], and subsequent oxidative dehydrocoupling with ZnCl_2_ and CuCl_2_. Selenophene precursor **10** itself was readily obtained in 68% yield from selenophene (**9**) in a one-pot procedure by successive lithiation with *n*-BuLi and quenching with trimethylsilyl chloride and iodine, respectively. We reacted biselenophene **11** with K_2_S as sulfur source and catalytic amounts of CuI in acetonitrile at 140 °C (vide supra) to afford TMS-protected DST **12** in 97% yield, which was subsequently deprotected with TBAF to parent DST **3** in 91% yield after purification. The trimethylsilyl-substituted precursor **12** was recently synthesized by Wang et al. from the corresponding dibromobiselenophene and benzene sulfonyl sulfide as sulfur source (50% yield) [27].

**Scheme 2:**
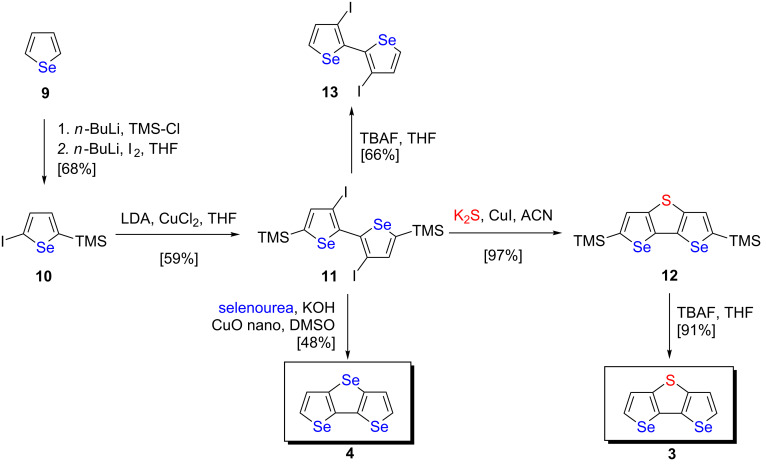
Synthesis of selenolotriacenes DST **3** and DSS **4**.

In parallel, TMS-protected iodinated biselenophene **11** was subjected to selenourea, copper oxide nanoparticles, and potassium hydroxide in DMSO to isolate diselenolo[3,2-*b*:2’,3’-*d*]selenophene (DSS, **4**) in 48% yield after purification (Scheme 2). Other selenation reagents such as selenium powder or disodium selenide were tested as well, but were not successful in order to giving increased yields of DSS **4**. In all reactions and optimization attempts, the TMS-groups were relatively quickly cleaved off from starting material **11** and dehalogenation was in parallel observed as competitive reaction pathway. Thus, mostly diiodobiselenophene **13** and 2,2’-biselenophene were isolated as main products. Independent reaction of deprotected diiodobiselenophene **13**, which was alongside prepared from TMS-biselenophene **11** by deprotection with TBAF in 66% yield, with selenourea and copper oxide nanoparticles surprisingly did not lead to any targeted DSS **4** in the attempted C–Se cross-coupling reaction.

The structures of the prepared novel selenolotriacenes **2**–**4** and known DTT **1** were characterized by means of NMR spectroscopy (Supporting Information File 1, Figures S1–S4), high-resolution mass spectrometry, and elemental analysis. In the ^1^H NMR spectra, the influence of the selenium atoms in triacenes DST **3** and DSS **4** results in substantial deshielding of the protons compared to bithiophene-based derivates **1** and **2**, which is in accordance with data for selenophene compared to thiophene [35].

### Single crystal X-ray structure analysis

**Figure 2:**
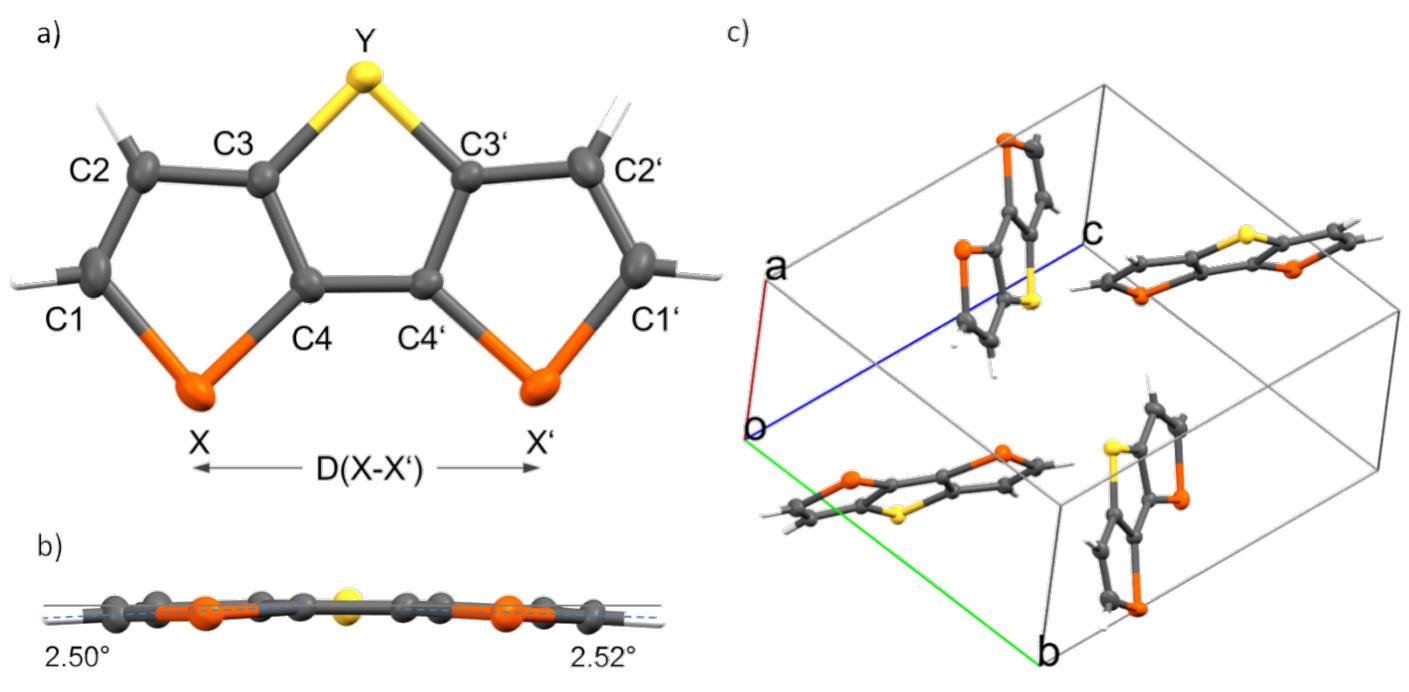
Single crystal X-ray structure analysis of selenolotriacene DST **3**, (a) individual molecule and atom numbering (top view); (b) side view. (c) Herringbone-type packing structure of the four molecules in the unit.

Single crystals of heterotriacenes DTS **2**, DST **3**, and DSS **4** suitable for X-ray structure analysis were obtained and details of the refinements are summarized in Tables S1-S3 (Supporting Information File 1). X-ray structure analysis of DTT **1** was already published by Brédas et al. [36–37]. Single crystals of DTS **2** and DSS **4** as very thin crystalline needles were obtainned by careful sublimation. Both heterotriacenes crystallized in the monoclinic space group *P*2_1_/*c* with 18 molecules in the unit cell (DTS **2**: a = 5.978(3), b = 29.005(11), c = 21.173(8) Å; α = 90°, β = 91.903(19)°, γ = 90°, *V* = 3669(3) Å^3^; DSS **4**: *a* = 6.108(3), *b* = 29.049(17), *c* = 21.949(11) Å; α = 90°, β = 91.815 (12)°, γ = 90°, *V* = 3892(3) Å^3^). The molecules in both crystals evidenced some rotational disorder. Single crystals of heterotriacene DST **3** were obtained by diffusion of *n*-hexane into a solution of DST **3** in dichloromethane. Triacene DST **3** crystallized in the monoclinic space group *P*2_1_/*n* with four equivalent molecules in the unit cell (*a* = 6.02748(19), *b* = 10.6662(3), *c* = 12.9279(4) Å; α = 90°, β = 96.747(3)°, γ = 90°) resulting in a unit cell volume of 825.38(4) Å^3^. The geometry of heterotriacene DST **3** is shown in the top and side view in Figure 2a and 2b, and for comparison purposes, bond lengths and angles from all four X-ray structure analyses of heterotriacenes **1**–**4** are summarized in Table 1.

**Table 1:** Bond distances, bond angles, and distances D of the outer heteroatoms obtained from the single crystal X-ray structure analysis of heterotriacenes **1** to **4**.

Hetero- triacene	Bond distance (Å) C_1_–C_2_/C_2_–C_3_/C_3_–C_4_/C_4_–C_4’_	Bond distance (Å) C_1_–X/X–C4/C_3_–Y	Angles (°) C_1_XC_4_/C_3_YC_3’_/XC_4_C_4’_	D(X–X’) (Å)	Angle (°) C_1_YC_1’_

DTT **1**^a^	1.36/1.42/1.38–9/1.42	1.73/1.72/1.74	91/90/137	3.94	105
DTS **2**	1.35–6/1.41/1.37–8/1.44	1.73/1.72–3/1.88–9	91/86/135	3.87	100
DST **3**	1.35/1.42–3/1.38/1.42	1.87–8/1.87/1.74	86.5/91.5/137	4.145	109
DSS **4**	1.34–5/1.41–2/1.36–8/1.43	1.88/1.88/1.89–91	86/87/134	4.08	104

^a^Data taken from reference [36–37].

The molecular volume in the crystals continuously increased from DTT **1** to DSS **4** (190.8 Å^3^, 203.8 Å^3^, 206.3 Å^3^, and 216.2 Å^3^) mostly due to the larger van der Waals radii of the selenium versus sulfur atoms (190 vs 180 pm) [38]. Bond distances and angles showed the expected differences between selenophene and thiophene rings: C–Se bonds are elongated by 0.16 to 0.17 Å compared to the C–S bonds and consequently the C–Se–C bond angles in selenophene rings are compressed to 86–87° compared to the C–S–C bond angle in the thiophene rings (90–91.5°) [39]. The C–Y “bridging” bonds always appeared elongated when compared to corresponding C–X bond distances. Remarkably, the distances between the external heteroatoms D(X–X’) are reduced by 0.07 Å in heteroacenes **2** and **4** containing Se atoms at the bridge position (Y) compared to **1** and **3**, while the inner bond distance (C_4_–C_4’_) barely change (0.01(2) Å). Although the molecular geometry of the heterotriacenes should be expected planar, a slight curvature of the π-system was found for DST **3**, whose α-carbon atoms are bent relative to the central thiophene plane by about 2.5 degrees (Figure 2b). This effect might be due to strong intermolecular π–π interactions in pairs of molecules (Figure 2b), because a completely flat geometry of the isolated molecule DST **3** (in the gas phase) was obtained from theoretical calculations (vide infra).

**Figure 3:**
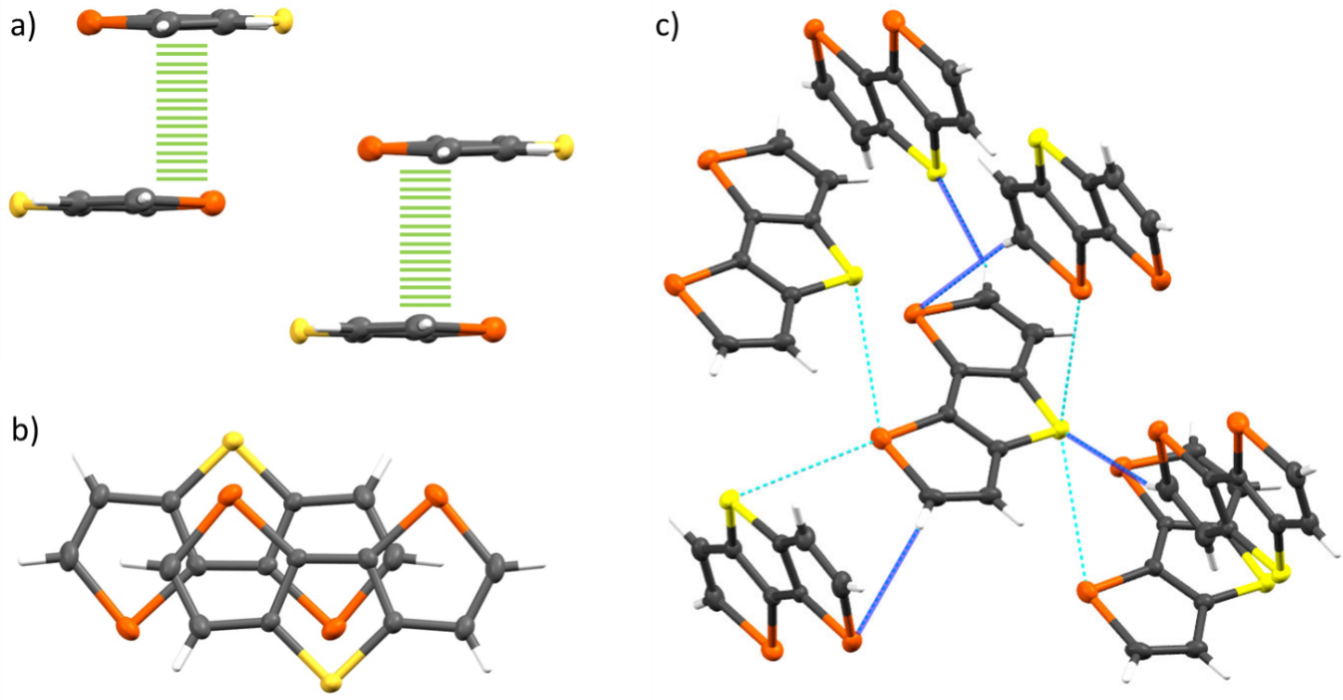
Single crystal X-ray structure analysis of selenolotriacene DST **3**: (a) partial overlap of stacked and displaced molecules leading to π–π interactions with distances down to 3.42 Å (side view); (b) and top view (64% molecular overlap). (c) Intermolecular interactions between heteroatoms and hydrogen-heteroatoms (labelled cyan and blue, respectively).

Molecules of DST **3** order in a typical herringbone fashion, where the terminal hydrogen atoms form hydrogen bond-like C–H heteroatom interactions (2.819 Å with S and 3.028 Å with Se) in a face-to-edge orientation (Figure 3c, Table S4a in Supporting Information File 1) [40]. We found as well several non-bonding S–Se contacts (3.644 Å) with four neighboring molecules in all crystallographic axes, which are slightly shorter than the sum of the van der Waals radii (3.70 Å), implying a 3-dimensional electronic coupling between the molecules of DST **3** in the crystal (Figure 3c, Table S2 in Supporting Information File 1). A similar situation has also been observed for DTS **2** (Figure S5, Table S1, and Table S5 in Supporting Information File 1) and DSS **4** (Figure S8, Table S3, and Table S6 in Supporting Information File 1).

In the case of DTT **1** only three non-bonding contacts between sulfur atoms in the b-axis direction were found which imply a 1-dimensional intermolecular electronic coupling in the molecular columns separated from each other by distances of 3.57 Å [36–37]. On the contrary, a much higher number of non-bonding contacts per molecule in all three space directions were identified for DTS **2** (10 contacts), DST **3** (8 contacts), and DSS **4** (14 contacts), respectively. Furthermore, in heterotriacenes **2**, **3**, and **4** we identified partial overlap of stacked and offset molecules leading to π–π interactions with distances as close as 3.42 Å for DST **3** (Figure 3a and b), 3.24 to 3.49 Å for DTS **2** (Figure S6a–c in Supporting Information File 1), and 3.28 to 3.58 Å for DSS **4** (Figure S8a–c in Supporting Information File 1). Interestingly, the symmetry of the formed dimers showed some differences: in DTT **1** the molecules overlap in a parallel orientation whereas in DST **3** an antiparallel orientation of the molecules in the dimer was found. The degree of overlap was determined to 73% and 64% for DTT **1** and DST **3**, respectively. Less degree of overlap (43–53% and 45–52%) and a mixture of both, parallel and antiparallel stacked dimers, were found in the X-ray structure analysis of heterotriacenes **2** (Figure S6b,c) and **4** (Figure S8b and S8c in Supporting Information File 1), respectively.

## XRD powder measurements

For completion, we performed XRD measurements on microcrystalline powders of all derivatives (Supporting Information File 1, Figure S9). At first glance, the stronger intensity of the signals for DTT **1** and DST **3** clearly evidences a higher crystallinity compared to triacenes DTS **2** and DSS **4**. XRD plots of heterotriacenes **1** to **3** obtained from the corresponding single crystal structure analysis were compared to the X-ray powder diffraction spectra (Figures S10, S11 and S12, in Supporting Information File 1). Whereas no correlation of the main peaks was found for DTT **1**, DTS **2** showed a better relationship between the powder and single crystal derived powder spectra. A very good correlation with almost no systematic error in peak positions can be clearly identified in the case of heterotriacene DST **3** (Figure S12, Supporting Information File 1) indicating a similar dominating crystalline phase in the microcrystalline powder and in the single crystal. Relevant signals at expected strong π–π intermolecular distances of 3.5–3.3 Å (2Θ = 25–26°), at offset π–π intermolecular distances of 4.1 Å (2Θ = 21.5°), and at herringbone intermolecular interaction distances of 8.2 Å (2Θ = 10.8°) were found and correlated with the Miller indices obtained in the X-ray single crystal structure analysis. XRD plots of DST **2** and DSS **4** showed strong diffusion scattering vs signal intensity which we assign to a high degree of amorphous phases. The crystallite sizes determined were quite similar for **1**, **2**, and **3** (66 nm, 76 nm, and 72 nm), respectively, except for DSS **4** which were smaller with 52 nm. The lack of correlation between the spectra for DTT **1** (Figure S10 in Supporting Information File 1) accounts for a completely different crystalline phase in the XRD vs the multicrystalline powder spectrum. Nevertheless, the high crystallinity observed in XRD measurements of heterotriacenes **1** and **3** rationalize their unexpected higher melting point compared to **2** and **4**.

### Quantum chemical calculations

**Figure 4:**
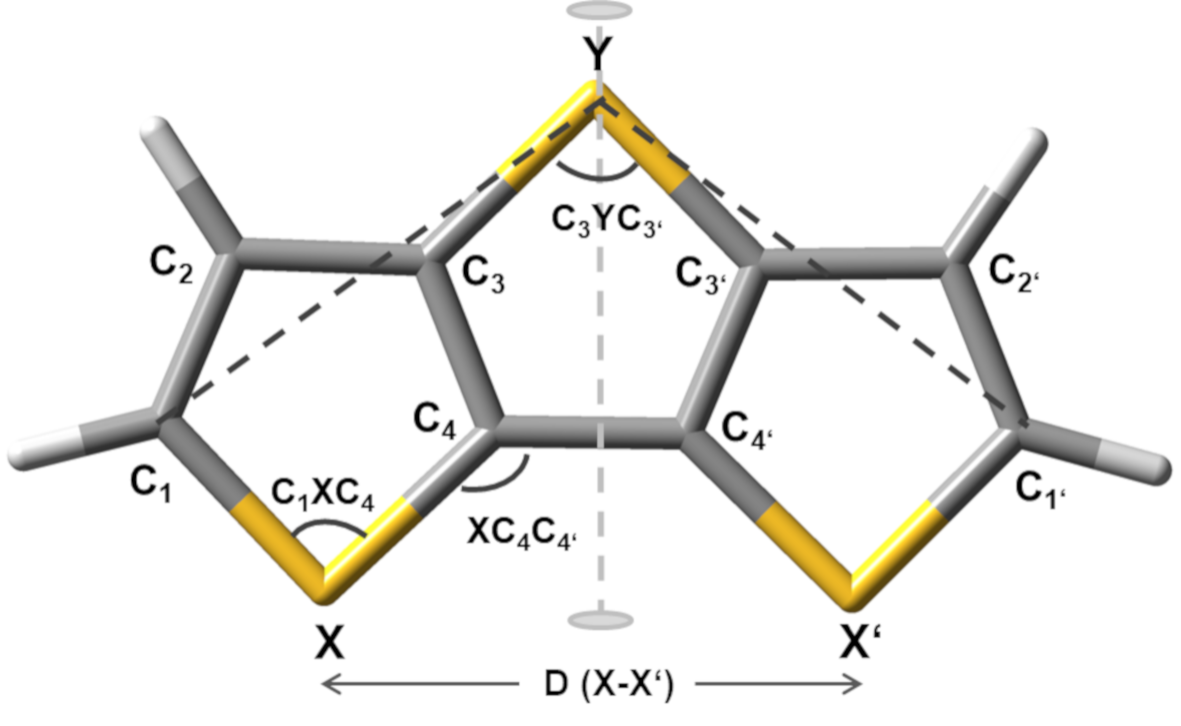
DFT quantum chemical calculated geometry of DTT **1** and general atom labelling for all heterotriacenes **1**–**4** discussed.

Quantum chemical DFT and TDDFT calculations (CAMB3LYP and B3LYP with the functional 6-31G^++^ (d,p)) were performed for the ground and excited state of heterotriacenes **1**–**4** in order to investigate their geometry and electronic properties. The optimized geometry of DTT **1** is shown in Figure 4, and most relevant corresponding bond distances and angles for all derivatives are summarized in Table 2. The comparative analysis of the alternating double-single bonds in the π-system of the heterotriacenes **1**–**4** evidenced only a slight increase of the interring bond (C_4_–C_4’_) for DTS **2** (1.43 Å) and consequently a smallest bite angle (C_1_YC_1’_ = 100°) despite the longest C–Se bond in the series (1.88 Å). The C-heteroatom distances vary for S (1.73–1.75 Å) to Se (1.85–1.88 Å) with the peculiarity that the longer distances in both cases correspond to the C_3_–Y bond. This was already observed in the X-ray structure analysis. The distances between the external heteroatoms D (X–X’) are reduced by introducing the bigger Se atom in the bridge position (Y) and in all cases are shorter than the ones obtained from the crystal structure analysis.

**Table 2:** DFT quantum chemical calculations on the geometry of heterotriacenes **1–4**.

Heterotriacene	Bond distance (Å) C_1_–C_2_/C_2_–C_3_/C_3_–C_4_/C_4_–C_4’_	Bond distance (Å) C_1_–X/X–C_4_/C_3_–Y	Angles (°) C_1_XC_4_/C_3_YC_3’_/XC_4_C_4’_	(Å) D(X–X’)	Angle (°) C_1_YC_1’_

DTT **1**	1.36/1.42/1.38/1.42	1.74/1.73/1.75	91/90/135	3.88	104
DTS **2**	1.36/1.42/1.38/1.43	1.74/1.73/1.88	91/87/134	3.84	100
DST **3**	1.36/1.43/1.38/1.42	1.88/1.85/1.75	87/90/136	4.10	108
DSS **4**	1.36/1.43/1.38/1.42	1.87/1.85/1.88	87/87/134	4.00	104

**Figure 5:**
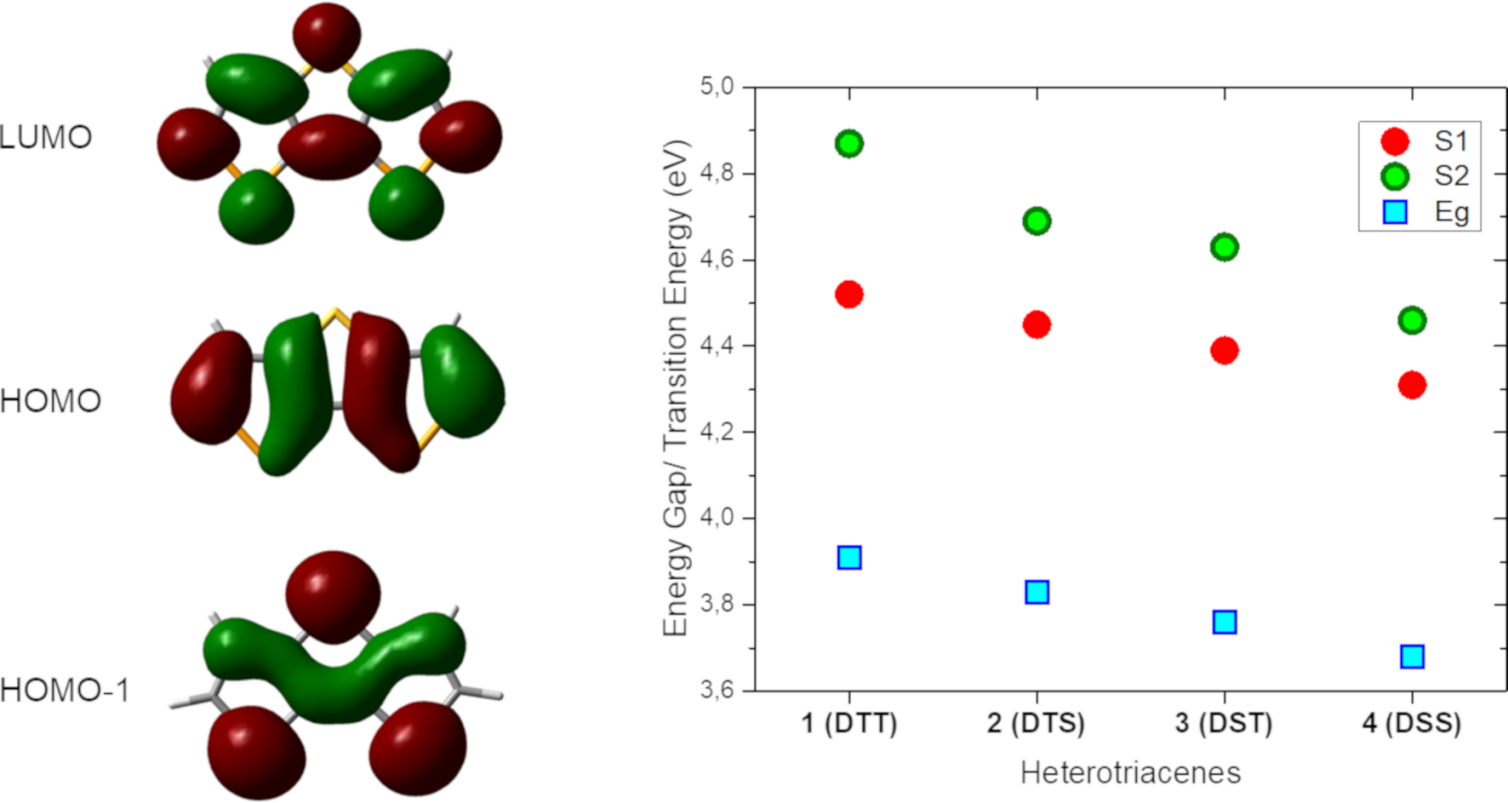
Representative electron density of frontier orbitals LUMO, HOMO, and HOMO-1 for heterotriacene DSS **4** (left). Energy of the first S_1_ (red dot) and second S_2_ (green dot) electronic transition calculated with TD-DFT and experimental energy gap (blue squares) of heterotriacenes **1** to **4** (right).

The analysis of the theoretical calculations gave also insight into the electronic properties of the heterotriacene series. The energies of the calculated frontier orbitals and electronic transitions are summarized in Table 3. In this respect, the energy of the HOMO slightly destabilizes from DTT **1** to DSS **4** in accordance to the decreasing aromatic character of the selenophene-based derivatives. A strong influence of the selenium atoms on the HOMO-1 and the LUMO can be observed (Figure 5, left and Table 3): the heavier selenium atoms gradually stabilize the LUMO and strongly destabilize HOMO-1. The calculated energy gap decreases from thiophene-based DTT **1** to selenium-containing derivative DSS **4** in accordance with the trend found for the experimentally determined optical energy gaps (vide supra).

**Table 3:** DFT and TDDFT quantum chemical calculations on heterotriacenes **1–4**.

Hetero- triacene	HOMO-1 [eV]	HOMO [eV]	LUMO [eV]	S_1_ [nm/eV] (f)	S_2_ [nm/eV] (f)	E_g_ [eV]

DTT **1**	−7.59	−7.23	−0.19	274/4.52 (0.38)	254/4.87 (0.17)	7.04
DTS **2**	−7.42	−7.22	−0.24	278/4.45 (0.34)	265/4.69 (0.17)	6.98
DST **3**	−7.39	−7.21	−0.30	283/4.39 (0.34)	268/4.63 (0.18)	6.91
DSS **4**	−7.26	−7.20	−0.36	288/4.31 (0.31)	278/4.46 (0.19)	6.84

**Figure 6:**
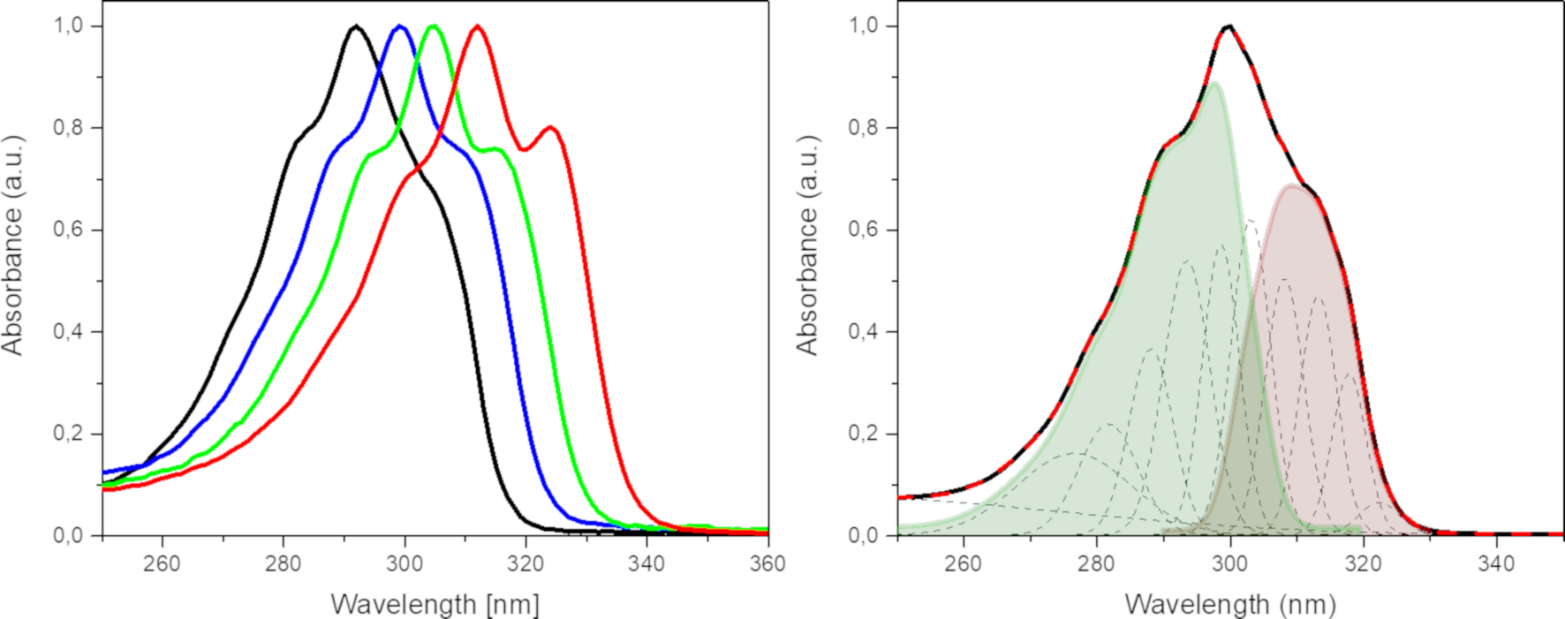
Normalized absorption spectra of heteroacenes DTT **1** (black line), DTS **2** (blue line), DST **3** (green line), and DSS **4** (red line) in dichloromethane (left). Gaussian deconvolution analysis of the absorption spectrum of DTT **1** (right): Gaussian deconvolution peaks (doted black lines), S_1_ transition (light red curve, balanced sum of the first 5 Gaussians), S_2_ transition (light green curve, balanced sum of Gaussians 6 to 10), and complete cumulative peak-fit (doted red line).

TDDFT calculations on heteroacenes **1**–**4** revealed the coexistence of two electronic transitions in a very narrow range of the spectrum: HOMO → LUMO transition, S_1_, whose transition dipole is oriented along the long-axis of the molecule and a HOMO-1 → LUMO transition, S_2_, whose transition dipole orients perpendicular to the long axis of the molecule (Table 3). In Figure 5 (right), the transition energies of S_1_ and S_2_ as well as the experimentally determined energy gaps are depicted for the heterotriacenes under investigation. The dependence of both transitions energies on the heteroatom character of the triacenes is shown. Both transitions gradually bathochromically shift from DTT **1** to DSS **4**, with stronger stabilization of the S_2_ transition which is coherent with the large atomic contribution from the heteroatoms to the involved molecular orbitals HOMO-1 and LUMO (vide supra). We can conclude that the theoretically calculated transitions S_1_ and S_2_ are reflected in the experimentally obtained absorption spectra (Figure 6) being responsible for the slightly different shape of their fine structure. The latter has been analyzed through Gaussian deconvolution of the absorption spectra and the two expected transitions for heterotriacene DTT **1** are shown (Figure 6, right).

### Optical properties

The optical properties of the four heterotriacenes were investigated by UV–vis and fluorescence spectroscopy in dichloromethane solution (Figure 6, left and Table 4). The absorption spectra in the series of DTT **1** to DSS **4** showed one main absorption band exhibiting vibronic fine structure according to the planar π-conjugated system. Gaussian deconvolution of the experimental spectra exemplarily shown for DDT **1** (Figure 6, right) evidenced the coexistence of two electronic transitions under the absorption curve in correlation with the theoretical calculations (vide infra). The absorption maxima are continuously red-shifted from DTT **1** to DSS **4** the more selenium atoms are present in the heteroacene (292–312 nm). This finding can be explained by the slightly lower aromaticity of the selenophene rings compared to thiophenes as a result from the slightly lower electronegativity (EN 2.55 vs 2.58) and significantly greater polarizability of selenium compared to sulfur atoms (P 3.77 Å^3^ vs 2.9 Å^3^) [41–43]. This effect is also obvious in a red-shift of the absorption maximum from 2,2’-bithiophene **5** (304 nm) to 2,2’-biselenophene **6** (328 nm) [44] as non-bridged counterparts of DTT **1**/DST **3** and DTS **2**/DSS **4**, respectively, which is explained in theoretical studies by a higher quinoidal character of the oligoselenophenes and a higher twisting barrier of the interring C–C bonds compared to oligothiophenes. The optical energy gaps, *E*_g_, are in accordance with the observed trend and decrease from 3.91 eV for DTT **1** to 3.67 eV for DSS **4** due to a stabilization of the HOMO energy level with increasing number of selenium atoms in the heteroacene (vide infra). The extinction coefficients are as well sensitive to the heteroatom in the bridge for pair DTT **1**/DTS **2** (26,800 to 22,100 L mol^−1^ cm^−1^) and DST **3**/DSS **4** (28,400 to 20,830 L mol^−1^ cm^−1^). No fluorescence was observed for each of the four heteroacenes DTT **1** to DSS **4** neither in DCM nor in THF.

**Table 4:** Thermal, optical, and electrochemical properties of heterotriacenes **1**–**4**.

Heterotriacene	Mp [°C]	λ_abs_ [nm]^a^	ε [L mol^−1^ cm^−1^]	*E*_g_^opt^ [eV]^b^	*E*_p_^ox^ [V]	HOMO [eV]^c^	LUMO [eV]^d^

DTT **1**	69.8	292	26800	3.91	0.94	−5.92	−2.01
DTS **2**	62.1	299	22100	3.83	0.84	−5.87	−2.04
DST **3**	120.6	305, 315	28400	3.76	0.82	−5.82	−2.06
DSS **4**	90.1	312, 324	20830	3.67	0.80	−5.80	−2.13

^a^Measured in dichloromethane solution (10^−4^ M). ^b^Estimated using the onset of the UV–vis spectrum in solution by *E*_g_^opt^ = 1240/λ_onset_. ^c^Estimated from the onset of the respective oxidation waves, Fc/Fc^+^ value set to −5.1 eV vs vacuum [45]. ^d^Determined from the optical band gap and HOMO.

### Electrochemical properties and electropolymerization

**Figure 7:**
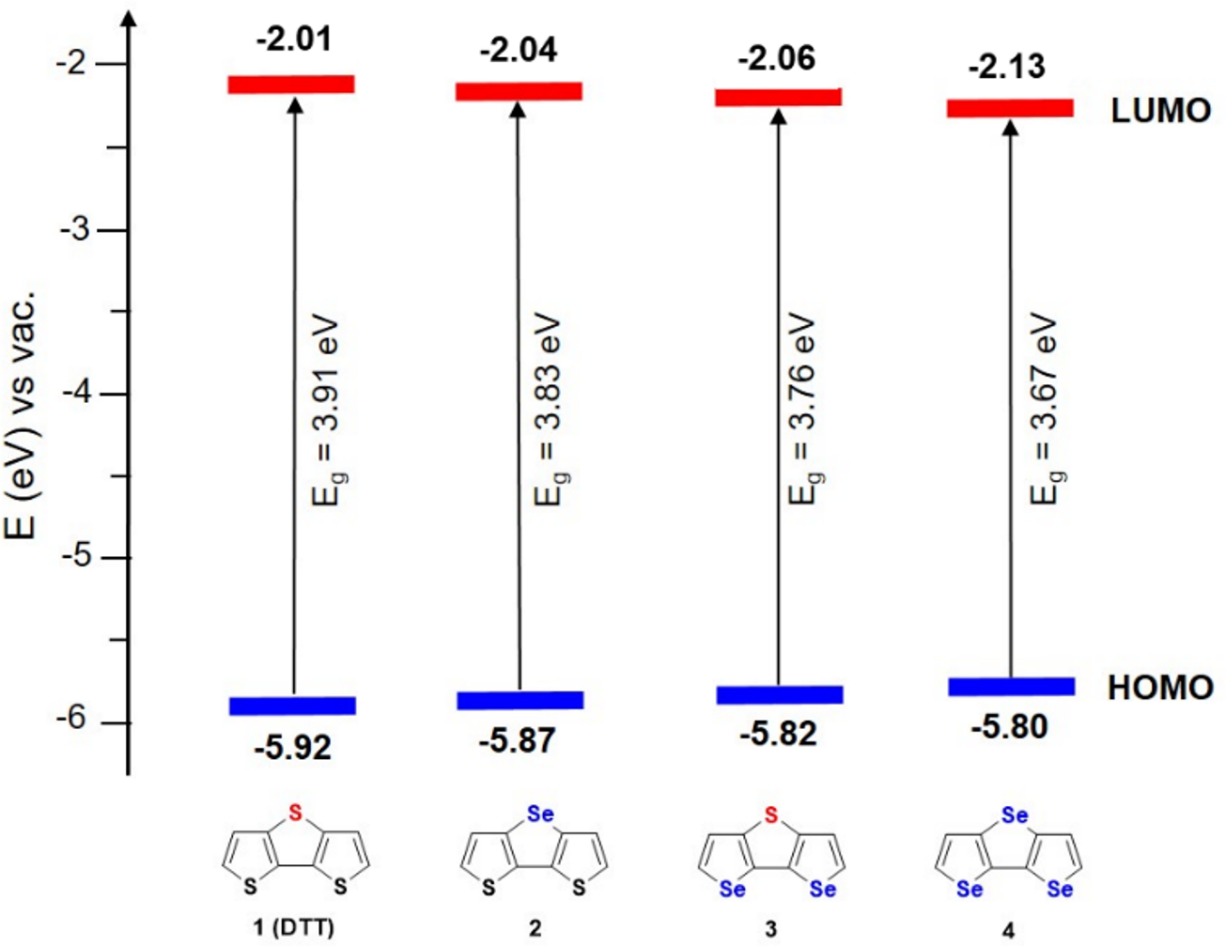
Energy diagram of the frontier molecular orbitals of heterotriacenes **1**–**4**.

The redox properties of the heterotriacenes **1**–**4** were investigated by means of cyclic voltammetry in the electrolyte tetrabutylammonium hexafluorophosphate (TBAPF_6_)/acetonitrile (Table 4, Figure S13 in Supporting Information File 1). The voltammogram of DDT **1** revealed one irreversible oxidation signal at 0.94 V (vs Fc^+^/Fc), which is in accordance to literature values [46]. Because selenophenes are slightly less aromatic than thiophenes with increasing number of selenium atoms a continuous decrease of the anodic peak potential was observed going from **2** (0.84 V) over **3** (0.82 V) to **4** (0.80 V). In comparison, dithienopyrrole (DTP), a corresponding nitrogen-bridged 2,2’-bithiophene, with a peak potential of 0.49 V, is much easier to oxidize due to the electron-rich character of the pyrrole ring [47]. The HOMO energy levels were determined from the onset of the oxidation wave and accordingly gradually decreased from **1** to **4** (−5.92 eV to −5.80 eV) (Table 4, Figure 7). Due to the absence of reduction waves in the cyclic voltammograms, the LUMO energy levels were calculated from *E*_g_^opt^ and the HOMO energy and decrease with increasing amount of selenium atoms in the heterotriacenes.

**Figure 8:**
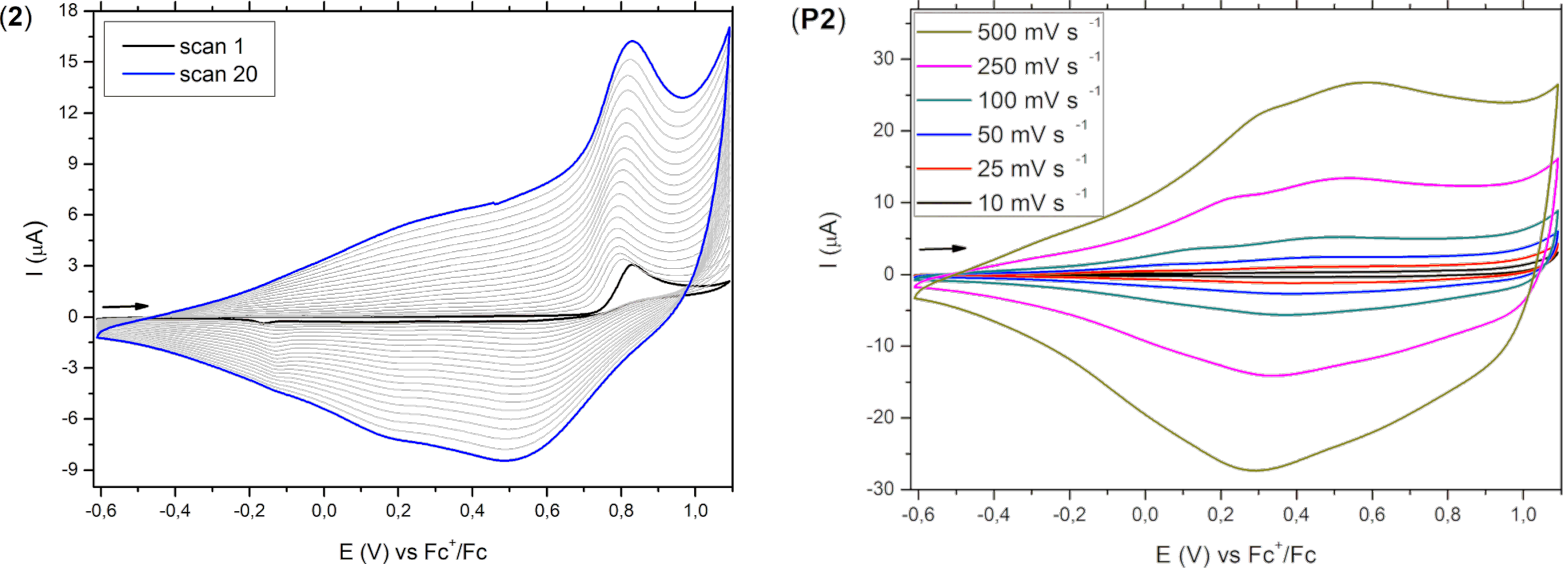
Multisweep voltammograms for the electrochemical polymerization of monomeric heterotriacene DST **2** in CH_2_Cl_2_/TBAPF_6_ (0.1 M) at a scan rate of 100 mV s^−1^ (left) and electrochemical characterization of the corresponding polymer P(DTS) **P2** in CH_2_Cl_2_/TBAPF_6_ (0.1 M) using different scan rates (right).

**Scheme 3:**
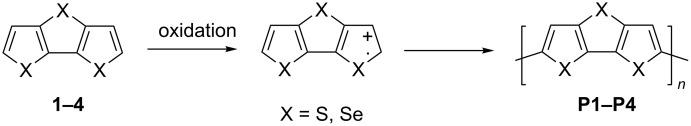
Oxidative polymerization of heterotriacenes **1**–**4** to corresponding conjugated polymers **P1–P4**.

Because of the structural similarity of heterotriacenes **1**–**4** to 2,2’-bithiophene and 2,2’-biselenophene, which can be oxidatively polymerized to polythiophenes [48–50] or polyselenophenes [51], respectively, we were interested in the electropolymerization of heterotriacenes **1**–**4** to the corresponding conjugated polymers **P1–P4**. Hence, monomers **1**–**4** were subjected to potentiodynamic polymerization in dichloromethane/TBAPF_6_ as electrolyte and the redox and optical properties of the obtained films were determined. Electropolymer P(DTT) **P1** has already been reported in literature and the findings agree well with our results [46,51]. In Figure 8, exemplarily the electropolymerization of heterotriacene DST **2** (left) and subsequent electrochemical characterization of polymer P(DTS) **P2** at various scan rates in a monomer-free electrolyte is shown (right). The other examples for **P1**, **P3**, and **P4** are shown in Supporting Information File 1, Figure S14. After the oxidation of the monomer in the first scan, polymerization starts by coupling of the emerging radical cations via the more reactive α-positions forming a film on the surface of the working electrode (Scheme 3). Calculations on radical cations of heterotriacenes **1–4** clearly showed that spin density is by far highest at the α- and low at the β-positions. Therefore, we assume that coupling and polymerization of the radical cations occurs via the α-positions leading to mostly linear conjugated systems without branching. In subsequent scans, broad cathodic and anodic signals emerged and with increasing number of cycles the respective currents continuously increased indicating the steady growth of polymer film. After 20 sweeps, homogeneous films of polymers **P1–P4** were obtained (observed by optical microscopy) which were then electrochemically and spectroelectrochemically characterized (Table 5 and Table S7 in Supporting Information File 1).

**Table 5:** Electrochemical properties of poly(heterotriacenes) **P1**–**P4** and film loss after conducting 30 scans in a monomer-free electrolyte solution.

Poly(heterotriacene)	*E*_pa_^a^ [V]	*E*_pc_^b^ [V]	*E*_onset_ [V]	HOMO [eV]	Film loss [%]^c^

P(DTT) **P1**	0.45	0.39	−0.18	−4.92	18
P(DTS) **P2**	0.51	0.37	−0.15	−4.95	19
P(DST) **P3**	^d^	0.33	−0.17	−4.93	8
P(DSS) **P4**	^d^	0.27	−0.12	−4.98	3
P(DTP) [33]	0.18	0.19	−0.54	−4.56	6

Potentials are referenced vs Fc^+^/Fc. ^a^*E*_pa_: anodic peak potential (scan rate 100 mV/s). ^b^*E*_pc_: cathodic peak potential (scan rate 100 mV/s). ^c^Determined as the difference of exchanged charges during the oxidation in scan 2 and scan 30, respectively. ^d^Could not be determined.

Cyclic voltammograms of polymers **P1–P4** in a monomer-free electrolyte showed broad and unstructured redox waves typical for conducting polymers reflecting the inhomogeneity of the material containing various electrophoric moieties due to variations in the (conjugated) chain length and conformational issues [52]. As well the relatively large shifts of the peak potentials with increasing scan rate, which are due to reduced diffusion of counter ions through the film, hinder the exact determination of redox potentials and trends among the derivatives of the series. Nevertheless, onset potentials, which reflect the starting transition between semiconducting and conducting state of the polymer, are indicative and for all four polymers **P1–P4** are located in the same range at −0.12 V to −0.18 V and vary only little. Therefore, the effect of the selenium atoms, which we saw for the oxidation of the corresponding monomers **1–4**, i.e., a lowering of the oxidation potential with increasing number of selenium atoms (vide supra), seems to be blurred for the polymers. Published data for P(DTT) **P1** is very similar showing an onset potential of −0.12 V vs Fc/Fc^+^ (calculated from 0.37 V vs Ag/AgCl) [46,52]. The redox characteristic of **P1–P4** is slightly more negative compared to the non-bridged counterparts, namely poly(bithiophene) [48–50] and poly(biselenophene) [51] (both show *E*_onset_ at ca. −0.0 V vs Fc/Fc^+^) indicating that the chalcogenide bridges do not much influence the electrochemical properties of the corresponding conjugated polymers. The related poly(dithienopyrrole) P(DTP) in contrast is more electron-rich and much easier to oxidize (*E*_onset_ = −0.54 V vs Fc/Fc^+^) [47]. Additionally, we evaluated the electrochemical stability of polymers **P1**–**P4.** After performing 30 sweeps, about 18–19% of the electroactivity was degraded for the bithiophene-based poly(heterotriacenes) P(DTT) **P1** and P(DTS) **P2** and only 3–8% for the biselenophene-based counterparts P(DST) **P3** and P(DSS) **P4** which is similar to P(DTP) [47] (Figure S15 in Supporting Information File 1).

The optical properties of polyheterotriacenes **P1**–**P4** were determined via spectroelectrochemistry using a previously described setup with a platinum working electrode and UV–vis–NIR spectra were recorded in reflectance mode [53]. At the beginning of the measurements a potential of −500 mV (vs Ag/AgCl) was applied in order to obtain the neutral polymer films without any oxidized parts. Then, the potentials were gradually increased until the oxidized polymers were obtained in their polaronic/bipolaronic states. In the neutral state, the most intense and broad bands of the π–π* transition showed absorption maxima in the range of 532 nm for P(DTT) **P1** to 478 nm for P(DSS) **P4** which is comparable to P(DTP) (524 nm) [47]. The deviation of the absorption of P(DTT) **P1** to the literature value (480 nm) [46] can most likely be attributed to differences in the polymerization procedures. The optical energy gaps have been determined from the onset absorptions and show decreasing values from P(DTT) **P1** (*E*_g_ = 1.79 eV) to **P2**–**P4** (*E*_g_ = 1.66–1.67 eV). The oxidized polymers gave as expected very broad and flat absorption bands from the visible to the NIR regime of the spectra (400–1600 nm) which is typical for conducting polymers, but hamper the determination of maxima (Table S7, Figure S16 in Supporting Information File 1).

## Conclusion

In summary, we presented the synthesis and characterization of novel selenolotriacenes DTS **2**, DST **3**, and DSS **4** in comparison to known DTT **1**, in which a varying number and sequence of fused thiophene and selenophene rings is implemented. For their preparation, efficient multistep synthesis routes with good overall yields based on recently published transition metal-catalyzed C–S and C–Se coupling/cyclization reactions in the crucial cyclization steps of iodinated bithiophene and biselenophene precursors. Heterotriacenes **1**–**4** turned out to be stable and well soluble systems, which allowed for the determination of thermal, optical, and electrochemical properties. By single crystal X-ray structure analysis the geometric structure and packing motifs of selenolotriacenes **2**–**4** were determined. Quantum chemical calculations allowed for a deeper understanding of the geometric and electronic structure of the heterotriacenes. The optoelectronic properties were determined and valuable structure–property relationships were deduced giving insight into the role of the number and relative position of the S and Se heteroatoms in the equally long fused conjugated triacenes. Electrooxidative polymerization of triacenes **1**–**4** led to corresponding conducting polymers **P1**–**P4**, which were electrochemically and spectroelectrochemically characterized and the properties compared to the non-bridged counterparts.

## Experimental

### Instruments and measurements

NMR spectra were recorded on a Bruker Avance 400 (^1^H NMR: 400 MHz, ^13^C NMR: 100 MHz), normally at 25 °C. Chemical shift values (δ) are expressed in parts per million using the solvent (^1^H NMR, δ_H_ = 7.26 and ^13^C NMR, δ_C_ = 77.0 for CDCl_3_) as internal standard. The splitting patterns are designated as follows: s (singlet), d (doublet), t (triplet), m (multiplet). Coupling constants (*J*) relate to proton-proton couplings. GC–MS measurements were performed on a Shimadzu GCMS-QP2010 SE instrument. Melting points were measured via differential scanning calorimetric measurements (DSC) on a Mettler Toledo DSC823e under argon atmosphere at a heating rate of 10 °C/min. Elemental analyses were performed on an Elementar Vario EL instrument. High resolution MALDI–MS was measured on a Fourier Transform Ion Cyclotron Resonance (FT-ICR) mass spectrometer solariX from Bruker Daltonics equipped with a 7.0 T superconducting magnet and interfaced to an Apollo II Dual ESI/MALDI source. Single crystals were analysed on a Bruker SMART APEX-II CCD diffractometer (λ(Mo Kα)-radiation, graphite monochromator, ω and 4 scan mode) and corrected for absorption using the SADABS program [53]. The structures were solved by direct methods and refined by a full-matrix least squares technique on F^2^ with anisotropic displacement parameters for non-hydrogen atoms. The hydrogen atoms were placed in calculated positions and refined within the riding model with fixed isotropic displacement parameters (U_ISO_(H) = 1.2Ueq(C)). All calculations were carried out using the SHELXL program package in Olex2 (v. 1.2.10) [54]. Crystallographic data have been deposited with the Cambridge Crystallographic Data Center: DTS **2** CCDC 1897412; DST **3** CCDC 1025419; DSS **4** CCDC 1898450. UV–vis measurements were carried out in dry DCM in 1 cm cuvettes and recorded on a Perkin Elmer UV/VIS/NIR Lambda 19 spectrometer. Cyclic voltammetry experiments were performed with a computer-controlled Autolab PGSTAT30 potentiostat in a three-electrode single compartment cell (3 mL). The platinum working electrode consisted of a platinum wire sealed in a soft glass tube with a surface of A = 0.785 mm^2^, which was polished down to 0.25 μm with Buehler polishing paste prior to use to guarantee reproducible surfaces. The counter electrode consisted of a platinum wire and the reference electrode was an Ag/AgCl reference electrode. All potentials were internally referenced to the ferrocene/ferricenium couple (Fc/Fc^+^). For the measurements, concentrations of 10^−3^ M of the electroactive species were used in freshly distilled and deaerated dichloromethane (Lichrosolv, Merck) purified with a Braun MB-SPS-800 and 0.1 M (*n*-Bu)_4_NPF_6_ (Fluka; recrystallized twice from ethanol). Spectroelectrochemical measurements of the polymer films were carried out in a 0.1 M solution of (*n*-Bu)_4_NPF_6_ in dry DCM. The applied setup has been described in the literature [55]. A platinum working electrode, a Ag/AgCl reference electrode, and a platinum sheet as the counter electrode were used and measurements were conducted in reflectance mode. During recording the UV–vis–NIR spectra, the applied potential was kept constant. Instrumental artefacts due to the change of the detector were removed and marked in the spectra. Quantum chemical calculations were performed with the Gaussian 09 package: DFT and TDDFT with the B3LYP and CAMB3LYP functional and 6-31++(d,p) basis-set [56].

### Materials

Iodine, zinc(II) chloride, copper(II) chloride, potassium hydroxide, chlorotrimethylsilane, copper(I) iodide, and potassium phosphate were purchased from Merck. Diisopropylamine, bis(dibenzylideneacetone)palladium(0), tetrabutylammonium fluoride, selenourea, and copper oxide nanoparticles were purchased from Sigma-Aldrich. *n*-Butyllithium in *n*-hexane (1.6 M) was purchased from Acros Organics, selenophene from TCI, 3-bromothiophene from Fluorochem, potassium thioacetate from Alfa Aesar, potassium sulfide from Caesar & Loretz, and 1,1’-bis(diphenylphosphino)ferrocene (dppf) from Frontier Scientific. Absolute tetrahydrofuran, dichloromethane, and toluene were provided from Sigma-Aldrich and purified using a Büchi MB SPS-800. Dimethyl sulfoxide, acetonitrile, and acetone were purchased from Merck and Sigma-Aldrich, purified, and dried by standard methods prior to use. All synthetic steps were carried out under an argon atmosphere and all glassware used for reactions was dried prior to use. Column chromatography was performed on glass columns packed with silica gel, Merck Silica 60, particle size 40–63 µm (Macherey-Nagel). Thin-layer chromatography was performed on aluminum plates, pre-coated with silica gel Merck Si60 F254. 3,3’-Diiodo-2,2’-bithiophene (**5**) [30] and 5,5-bis(trimethylsilyl)-3,3’-diiodo-2,2’-bithiophene (**6**) [31] were prepared according to literature procedures.

### Synthesis

**2,6-Bis(trimethylsilyl)dithieno[3,2-*b*:2',3'-*d*]thiophene (7)** [20]. To a solution of 3,3’-diiodo-5,5’-bis(trimethylsilyl)-2,2’-bithiophene (**6**, 500 mg, 0.89 mmol) in dry acetonitrile (7 mL) was added copper(I) iodide (17 mg, 89 µmol, 10 mol %) and dipotassium sulfide (196 mg, 1.8 mmol) at rt. The mixture was heated to 140 °C and stirred for 16 hours. After cooling to rt, the reaction was quenched with water and the resulting mixture was extracted three times with diethyl ether. The combined organic layer was dried over Na_2_SO_4_ and concentrated under vacuum. The residue was purified by column chromatography (SiO_2_, petroleum ether) to give DTT (**7**) as a white solid (0.22 g, 0.65 mmol, 73%). Mp 94.6 °C (DSC); ^1^H NMR (CDCl_3_) δ (ppm) = 7.34 (s, 2H), 0.37 (s, 18H); ^13^C NMR (CDCl_3_) δ (ppm) 144.2, 142.5, 135.6, 127.1, 0.0; anal. calcd for C, 49.41; H, 5.88; S, 28.24; found: C, 48.65; H, 5.64; S, 29.18. The analytical data are in accordance with literature [32].

**Dithieno[3,2-*b*:2',3'-*d*]thiophene (DTT, 1)** prepared from **7**. To a solution of 2,6-bis(trimethylsilyl)dithieno[3,2-*b*:2',3'-*d*]thiophene (**7**, 92 mg, 0.27 mmol) in THF (2 mL) a solution of tetrabutylammonium fluoride trihydrate (184 mg, 0.6 mmol) in 1 mL THF was added. The mixture was stirred for 1.5 hours, filtrated, and concentrated under vacuum. The crude product was purified by column chromatography (SiO_2_, petroleum ether) to afford DTT **1** as a white solid (48 mg, 0.245 mmol, 91%). Mp 69.6 °C (DSC); ^1^H NMR (CDCl_3_) δ (ppm) 7.36 (d, ^3^*J* = 5.2 Hz, 2H), 7.29 (d, ^3^*J* = 5.2 Hz, 2H); ^13^C NMR (CDCl_3_) δ (ppm) 141.7, 131.0, 126.0, 120.9; anal. calcd for C, 48.95; H, 2.05; S, 49.00; found: C, 49.06; H, 2.10; S 49.24. The analytical data are in accordance with literature [57].

**Dithieno[3,2-*b*:2',3'-*d*]thiophene (DTT, 1)** prepared from **5**. To a solution of 3,3'-diiodo-2,2'-bithiophene (**5**, 500 mg, 1.2 mmol) in dry acetonitrile (14 mL) was added copper(I) iodide (23 mg, 0.12 mmol, 10 mol %) and dipotassium sulfide (264 mg, 2.4 mmol) at rt. The mixture was heated to 140 °C and stirred for 16 hours. After cooling to rt, the reaction was quenched with water and the resulting mixture was extracted three times with diethyl ether. The combined organic layer was dried over Na_2_SO_4_ and concentrated under vacuum. The residue was purified by column chromatography (SiO_2_, petroleum ether) to provide DTT **1** as a white solid (152 mg, 0.8 mmol, 66%). The analytical data was the same as described above.

**Selenolo[3,2-*b*:4,5-*b*’]dithiophene (2).** To a stirred solution of 3,3'-diiodo-2,2'-bithiophene (**5**, 200 mg, 0.48 mmol) and selenourea (118 mg, 0.96 mmol) in dry dimethyl sulfoxide (1.5 mL) at rt was added copper(I) oxide nanoparticles (4 mg, 48 µmol, 10 mol %) followed by potassium hydroxide (54 mg, 0.96 mmol). The mixture was heated at 80 °C for 20 hours, before a second portion of selenourea (118 mg, 0.96 mmol), copper(I) oxide (4 mg, 48 µmol, 10 mol %), and potassium hydroxide (54 mg, 0.96 mmol) was added. After stirring at 80 °C for another 20 hours, the reaction mixture was cooled to rt and a 1:1 mixture of dichloromethane/water was added. The combined organic extracts were collected, dried with anhydrous MgSO_4_ and concentrated under vacuum. The crude product was purified by column chromatography (SiO_2_, petroleum ether) and the product-enriched fractions were further purified by HPLC (*n*-hexane/CH_2_Cl_2_ 8:2) to afford the desired heterotriacene DTS **2** as a white solid (59 mg, 0.24 mmol, 51%). Mp 62.1 °C (DSC); ^1^H NMR (CDCl_3_) δ (ppm) 7.34 (d, ^3^*J* = 5.2 Hz, 2H), 7.33 (d, ^3^*J* = 5.2 Hz, 2H); ^13^C NMR (CDCl_3_) δ (ppm) 139.2, 132.4, 125.5, 123.7; anal. calcd for C, 39.51; H, 1.66; S, 26.37; found: C, 39.51; H, 1.63; S: 26.18. HRMS (APCI) *m*/*z*: [M^+^] calcd for C_8_H_4_S_2_Se, 243.89129; found, 243.89155; δ*m*/*m* = 1.07 ppm.

**5-Iodo-2-(trimethylsilyl)selenophene (10).** Selenophene (**9**, 2.00 g, 15 mmol) was dissolved under argon in dry THF (11 mL) and *n*-BuLi (1.6 M in hexane, 9.5 mL, 15 mmol) was added dropwise at −78 °C. The milky solution was stirred at −78 °C for 45 min. Chlorotrimethylsilane (2 mL, 16 mmol) was added and the mixture was stirred for one more hour. Then, another portion of *n*-BuLi (1.6 M, 10 mL, 16 mmol) was added. After stirring for one hour at −78 °C, a solution of elemental iodine (3.8 g, 15 mmol) in THF (8 mL) was added dropwise at −65 to −55 °C within 15 min. The solution was stirred for another hour at rt and remaining iodine was reduced with a sodium thiosulfate solution (10 mL). The mixture was quenched with water and extracted three times with diethyl ether. The organic layers were combined, dried over MgSO_4_, and concentrated under vacuum. The brown crude product was purified by column chromatography (SiO_2_; petroleum ether) to obtain pure selenophene **10** as a light yellow liquid (3.4 g, 10.3 mmol, 68%); ^1^H NMR (CDCl_3_) δ (ppm) 7.54 (d with ^77^Se-satellites, ^3^*J*_Se-H_ = 11.6 Hz, ^3^*J*_H-H_ = 3.6 Hz, 1H), 7.53 (d with ^77^Se-satellites, ^3^*J*_Se-H_ = 11.6 Hz, ^3^*J*_H-H_ = 3.6 Hz, 1H), 0.29 (s, 9H); ^13^C NMR (CDCl_3_) δ (ppm) 156.6, 141.8, 138.0, 79.9, 0.4; HRMS (APCI) *m*/*z*: [M^+^] calcd for C_7_H_11_ISeSi, 329.88343; found, 329.88409; δ*m*/*m* = 2.0 ppm.

**3,3’-Diiodo-5,5’-bis(trimethylsilyl)-2,2’-biselenophene (11).** *n*-BuLi (1.6 M in hexane, 4.6 mL, 7.3 mmol) was added dropwise to a solution of diisoproylamine (1.2 mL, 8.6 mmol) in dry THF (4 mL) at 0 °C and stirred for one hour. 5-Iodo-2-(trimethylsilyl)selenophene (**10**, 2.0 g, 6.1 mmol) was dissolved in dry THF (7.5 mL) and the LDA solution was added dropwise within 30 min. The mixture was stirred at −78 °C for 1.5 hours after complete addition of LDA and then a solution of zinc(II) chloride (1.0 g, 7.3 mmol) dissolved in 5.6 mL dry THF was added. After stirring for one hour at 0 °C, copper(II) chloride (986 mg, 7.3 mmol) was added in one portion and the resulting mixture was stirred at −78 °C for 3 hours, then at rt for 18 hours. The solvent was removed under reduced pressure. The crude product was purified by column chromatography (SiO_2_; petroleum ether) to obtain pure biselenophene **11** (1.47 g; 2.2 mmol, 59%) as a pale yellow solid. Mp 107.1 °C (DSC); ^1^H NMR (CDCl_3_) δ (ppm) 7.49 (s, with ^77^ Se-satellites, ^3^*J*_Se-H_ = 6.0 Hz, 2H), 0.34 (s, 18H); ^13^C NMR (CDCl_3_) δ (ppm) 153.4, 146.2, 144.33, 87.4, 0.2; anal. calcd for C, 25.62; H 3.07; found: C, 25.77; H: 2.89; HRMS (APCI) *m*/*z*: [M^+^] calcd for C_14_H_20_I_2_Se_2_Si_2_, 657.75199; found, 657.75033; δ*m*/*m* = 2.52 ppm.

**2,6-Bis(trimethylsilyl)bisselenolo[3,2-*b*:2',3'-*d*]thiophene (12).** To a solution of 3,3’-diiodo-5, 5’-bis(trimethylsilyl)-2,2’-biselenophene (**11**, 514 mg, 0.78 mmol) in dry and well-degassed acetonitrile (15 mL) copper(I) iodide (30 mg, 0.16 mmol, 20 mol %) and dipotassium sulfide (346 mg, 3.14 mmol) was added at rt. The mixture was heated to 140 °C and stirred for 20 hours. After cooling to rt, the reaction was quenched with water and the resulting mixture was extracted three times with diethyl ether. The combined organic layer was dried over MgSO_4_ and concentrated under vacuum. The residue was purified by column chromatography (alumina, petroleum ether) to obtain biselenolothiophene **12** as an orange solid (331 mg, 0.76 mmol, 97%). Mp 111.7 °C (DSC); ^1^H NMR (CDCl_3_) δ (ppm) 7.63 (s, with ^77^Se-satellites, ^3^*J*_Se-H_ = 6.8 Hz, 2H), 0.34 (s, 18H); ^13^C NMR (CDCl_3_) δ (ppm) 149.4, 145.2, 138.3, 129.4, 0.3; anal. calcd for C, 38.70; H, 4.64; S, 7.38; found: C, 38.60; H, 4.45; S, 7.49. The analytical data are in accordance with literature [27].

**Bisselenolo[3,2-*b*:2',3'-*d*]thiophene (DST, 3).** To a solution of 2,6-bis(trimethylsilyl)bisselenolo[3,2-*b*:2',3'-*d*]thiophene (**12**, 153 mg, 0.35 mmol) in THF (4 mL) was added tetrabutylammonium fluoride trihydrate (391 mg, 1.23 mmol) in 2 mL THF. The mixture was stirred for 2 hours, then filtrated and concentrated under vacuum. The crude product was purified by column chromatography (SiO_2_, petroleum ether) to afford bisselenolothiophene **3** as a lightly yellow solid (94 mg, 0.32 mmol, 91%). Mp 120.5 °C (DSC); ^1^H NMR (CDCl_3_) δ (ppm) 7.95 (d with ^77^Se-satellites, ^2^*J*_Se-H_ = 48.6 Hz, ^3^*J*_H-H_ = 5.7 Hz, 2H), 7.53 (d with ^77^Se-satellites, ^3^*J*_Se-H_ = 5.8Hz, ^3^*J*_H-H_ = 5.6 Hz, 2H); ^13^C NMR (CDCl_3_) δ (ppm) 142.7, 133.9, 129.7, 123.4. anal. calcd for C, 33.12; H, 1.36; S, 11.05; found: C, 33.70; H, 1.52; S, 11.64. HRMS (APCI) *m/z*: [M^+^] calcd for C_8_H_4_SSe_2_, 291.83583; found, 291.83625; δ*m*/*m* = 1.1 ppm.

**Bisselenolo[3,2-*b*:2',3'-*d*]selenophene (DSS, 4).** To a stirred solution of 3,3’-diiodo-5,5’-bis(trimethylsilyl)-2,2’-biselenophene (**11**, 100 mg, 0.15 mmol) and selenourea (28 mg, 0.23 mmol) in dry dimethyl sulfoxide (0.8 mL) under argon at rt was added copper oxide nanoparticles (1.2 mg, 10 mol %) followed by potassium hydroxide (26 mg, 0.46 mmol). The mixture was heated at 80 °C for 18 hours, cooled to rt, and a 1:1 mixture of dichloromethane/water was added. The combined organic extracts were collected, dried with anhydrous MgSO_4_, and concentrated under vacuum. The crude product was purified by column chromatography (SiO_2_, deactivated with 3% triethylamine, petroleum ether) to afford bisselenoloselenophene **4** as a lightly grey solid (25 mg, 70 µmol, 48%). Mp 90.1 °C (DSC); ^1^H NMR (CDCl_3_) δ (ppm) 7.94 (d with ^77^Se-satellites, ^2^*J*_Se-H_ = 48.4 Hz, ^3^*J*_H-H_ = 5.6 Hz, 2H), 7.56 (d with ^77^Se-satellites, ^3^*J*_Se-H_ = 5.6 Hz, ^3^*J*_H-H_ = 5.6 Hz, 2H); ^13^C NMR (CDCl_3_) δ (ppm) 140.7, 135.5, 129.6, 126.1; HRMS (APCI) *m*/*z*: [M^+^] calcd for C_8_H_4_Se_3_, 337.78158; found, 337.782251; δ*m*/*m* = 2.75 ppm.

**3,3’-Diiodo-2,2’-biselenophene (13).** To a stirred solution of 3,3’-diiodo-5,5’-bis(trimethylsilyl)-2,2’-biselenophene (**11**, 400 mg, 0.61 mmol) in THF (7 mL) at 0 °C under argon was added dropwise tetrabutylammonium fluoride trihydrate (400 mg, 1.3 mmol) in 1 mL THF. The mixture was warmed to rt and stirred for 1.5 hours. At the end of the reaction, the mixture was filtrated and concentrated under vacuum. The crude product was purified by column chromatography (SiO_2_, *n*-hexane/DCM 10:1) to afford biselenophene **13** as a white solid (202 mg, 0.4 mmol, 66%). ^1^H NMR (CDCl_3_) δ (ppm) 8.08 (d with ^77^Se-satellites, ^2^*J*_Se-H_ = 44 Hz, ^3^*J*_H-H_ = 5.8 Hz, 2H); 7.36 (d with ^77^Se-satellites, ^3^*J*_Se-H_ = 12 Hz, ^3^*J*_H-H_ = 5.8 Hz, 2H); ^13^C NMR (CDCl_3_) δ (ppm) 141.7, 138.4, 135.1, 86.6. HRMS (APCI): *m*/*z*: [M^+^] calcd for C_8_H_4_Se_3_, 513.67274; found, 513.67374; δ*m*/*m* = 1.6 ppm.

## Supporting Information

File 1 Additional spectral and crystallographic data.
