# Supplementary material for: Selenophene-containing heterotriacenes by a C–Se coupling/cyclization reaction
Source: Beilstein J Org Chem. 2019 Jun 24;15:1379–93. doi: 10.3762/bjoc.15.138 (PMC6604749; doi:10.3762/bjoc.15.138)
Supplement: File 1 — Additional spectral and crystallographic data. [file Beilstein_J_Org_Chem-15-1379-s001.pdf]

BEILSTEIN JOURNAL OF ORGANIC CHEMISTRY

# Supporting Information

for

# Selenophene-containing heterotriacenes by a C–Se coupling/ cyclization reaction

Pierre-Olivier Schwartz, Sebastian Förtsch, Astrid Vogt, Elena Mena-Osteritz and Peter Bäuerle

*Beilstein J. Org. Chem.* **2019,** *15,* 1379–1393. doi:10.3762/bjoc.15.138

# Additional spectral and crystallographic data

License and Terms: This is a supporting information file under the terms of the Creative Commons Attribution License (http://creativecommons.org/licenses/by/4.0). Please note that the reuse, redistribution and reproduction in particular requires that the authors and source are credited. The license is subject to the *Beilstein Journal of Organic Chemistry* terms and conditions: (https://www.beilstein-journals.org/bjoc)

## $^{1}H$ and $^{13}C$ NMR spectra of the target heterotriacenes 1–4

Dithieno[3,2-*b*:2',3'-*d*]thiophene (**1**, DTT)

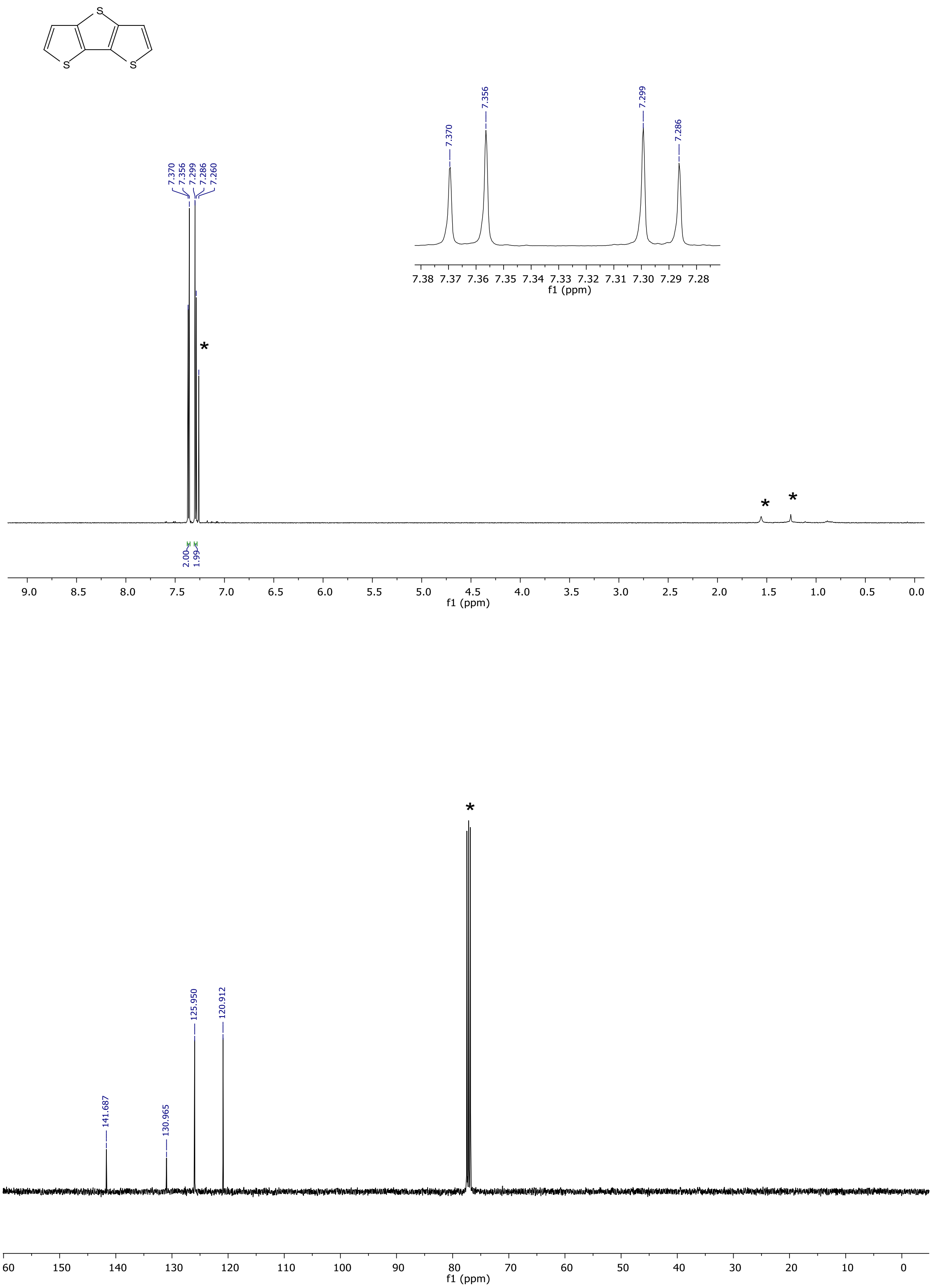


**Figure S1:** $^{1}H$ (top) and $^{13}C$ NMR (bottom) spectrum of heterotriacene **1** in $CDCl_3$. *= residual solvent signals

Selenolo[3,2-*b*:4,5-*b′*]dithiophene (**2**, DTS).

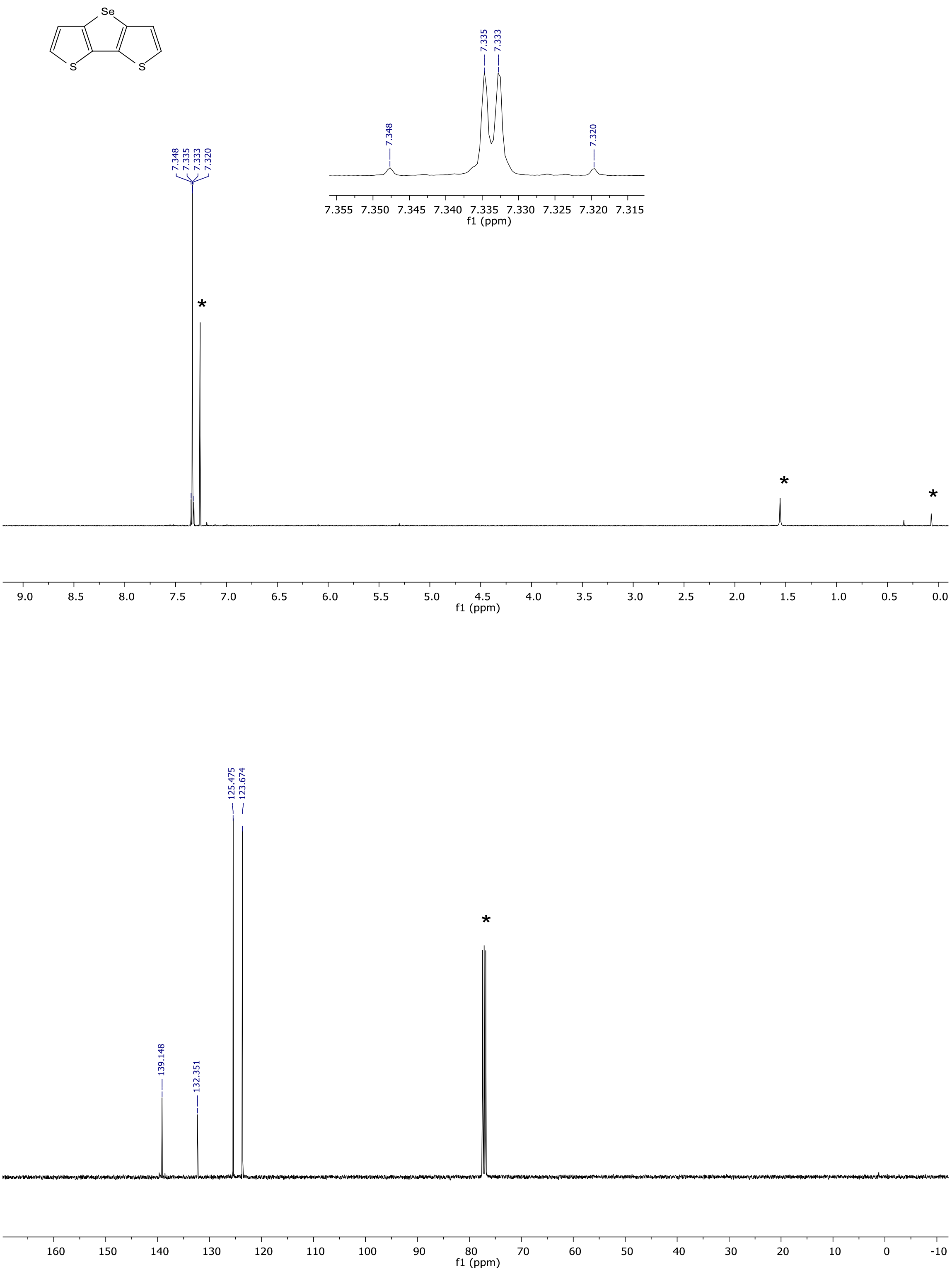


**Figure S2:** $^{1}H$ (top) and $^{13}C$ NMR (bottom) spectrum of heterotriacene **2** in $CDCl_3$. *= residual solvent signals

Bisselenolo[3,2-*b*:2',3'-*d*]thiophene (**3**, DST)

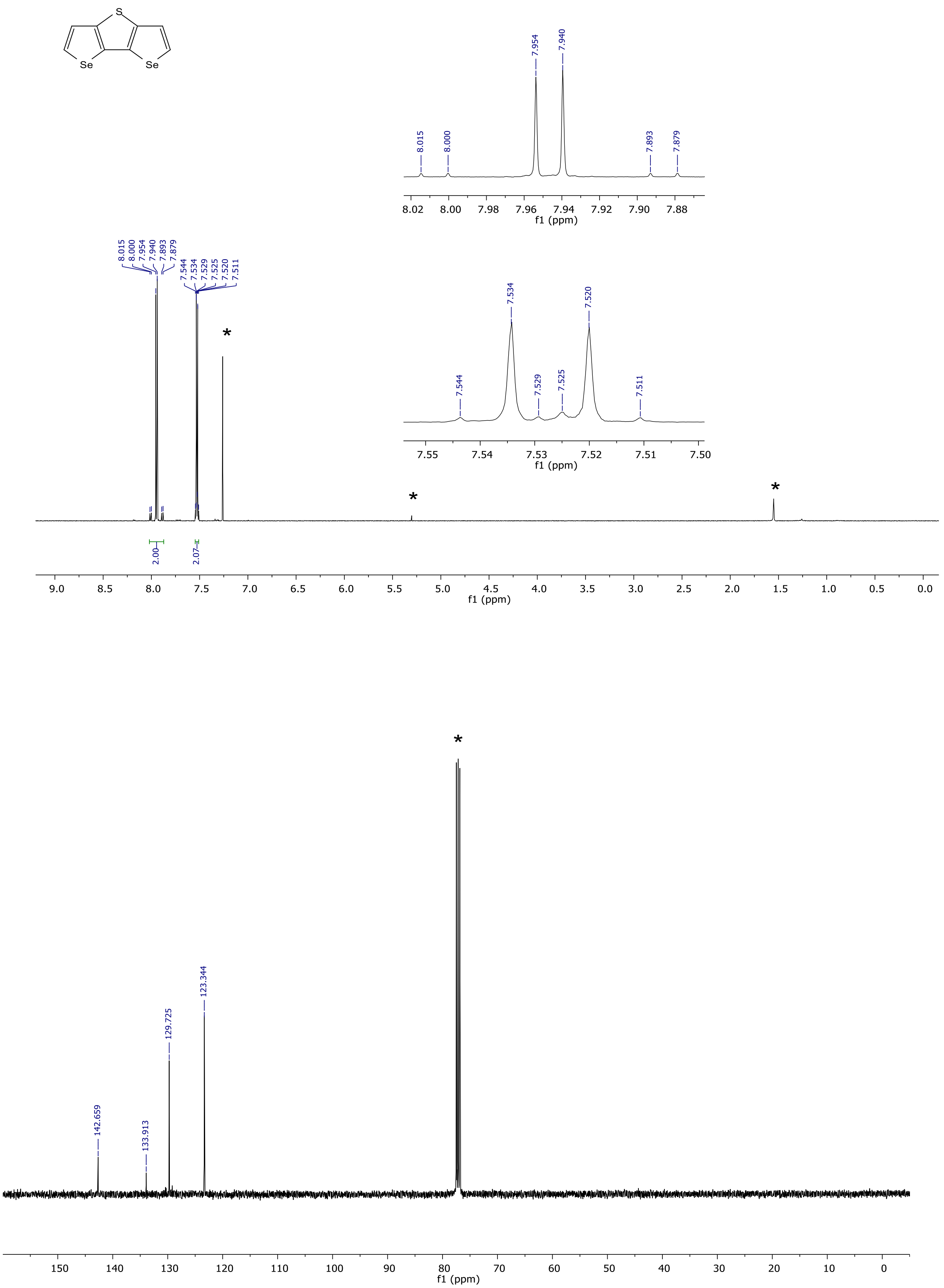


**Figure S3:** $^{1}$H (top) and $^{13}$C NMR (bottom) spectrum of heterotriacene **3** in $CDCl_3$. *= residual solvent signals

Bisselenolo[3,2-*b*:2',3'-*d*]selenophene (**4**, DSS)

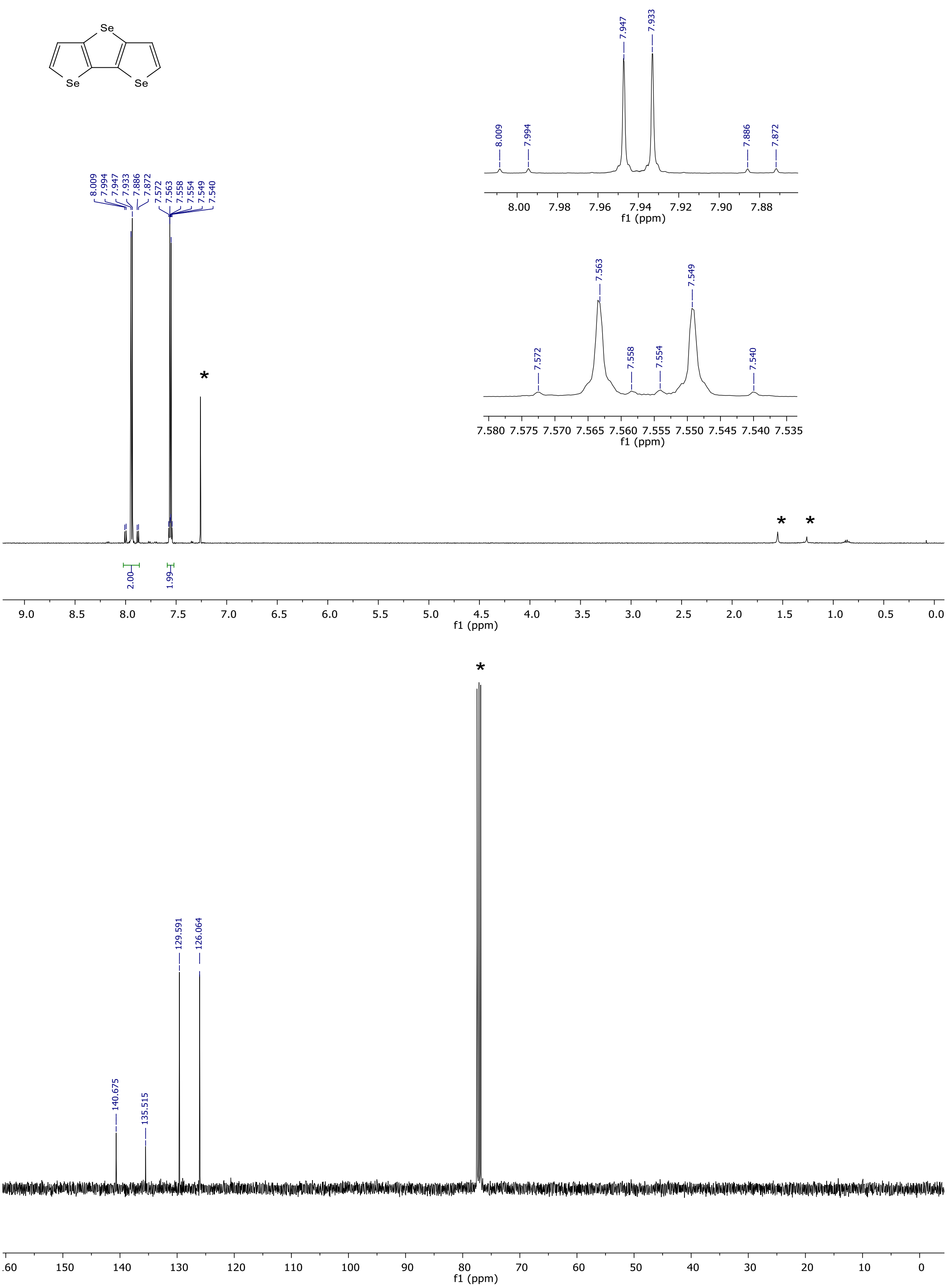


**Figure S4:** $^{1}H$ (top) and $^{13}C$ NMR (bottom) spectrum of heterotriacene **4** in in $CDCl_3$. *= residual solvent signals

## Single crystal X-ray structure analysis

**Table S1**: X-ray structure analysis data of heterotriacene DTS **2**.

| | | |
|---|---|---|
| Identification code | DTS **2** | CCDC 1897412 |
| Empirical formula | $C_{36}H_{18}S_9Se_{4.5}$ | |
| Formula weight | 1094.36 | |
| Temperature/K | 293.15 | |
| Crystal system | monoclinic | |
| Space group | $P2_1/c$ | |
| *a*/Å | 5.978(3) | |
| *b*/Å | 29.005(11) | |
| *c*/Å | 21.173(8) | |
| α/° | 90 | |
| β/° | 91.903(19) | |
| γ/° | 90 | |
| Volume/Å$^3$ | 3669(3) | |
| Z | 4 | |
| $\rho_{calc}$g/cm$^3$ | 1.981 | |
| μ/mm$^{-1}$ | 5.037 | |
| F(000) | 2124.0 | |
| Crystal size/mm$^3$ | 0.164 × 0.064 × 0.064 | |
| Radiation | MoKα (λ = 0.71073) | |
| 2Θ range for data collection/° | 4.098 to 61.022 | |
| Index ranges | -8 ≤ h ≤ 8, -41 ≤ k ≤ 41, -30 ≤ l ≤ 30 | |
| Reflections collected | 125755 | |
| Independent reflections | 11219 [$R_{int}$ = 0.0445, $R_{sigma}$ = 0.0221] | |
| Data/restraints/parameters | 11219/935/706 | |
| Goodness-of-fit on F$^2$ | 1.276 | |
| Final R indexes [I>=2σ (I)] | $R_1$ = 0.0557, $wR_2$ = 0.0977 | |
| Final R indexes [all data] | $R_1$ = 0.0632, $wR_2$ = 0.0998 | |
| Largest diff. peak/hole / e Å$^{-3}$ | 0.72/-0.76 | |

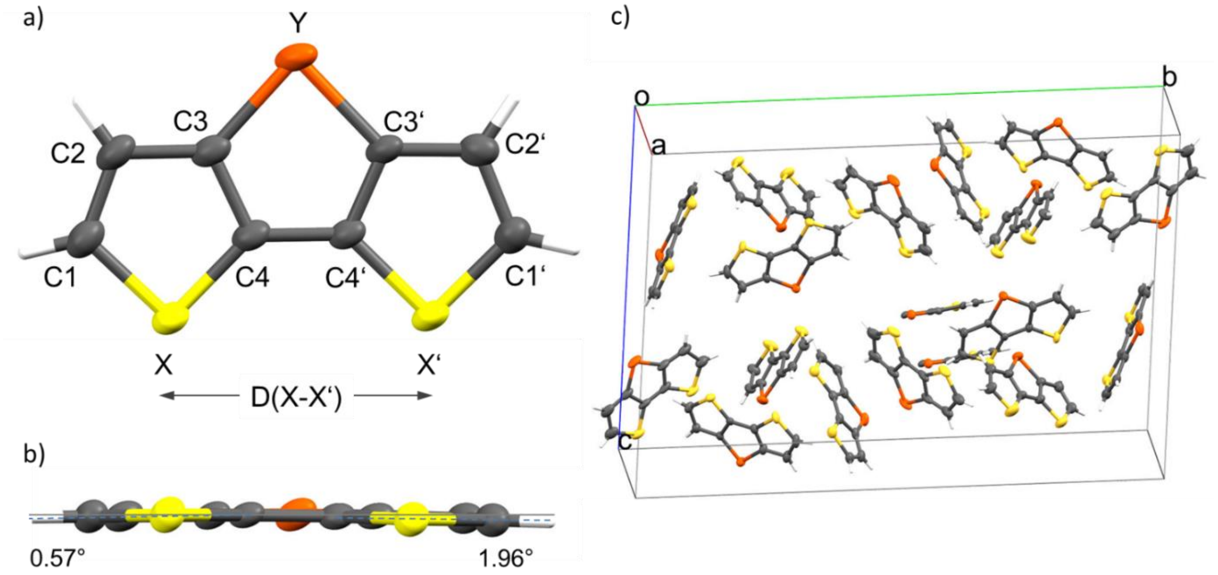


**Figure S5:** Single-crystal X-ray structure analysis of selenolotriacene DTS **2**, (a) individual molecule and atom numbering (top view); (b) side view. (c) Herringbone-type packing structure of the molecules in the unit cell.

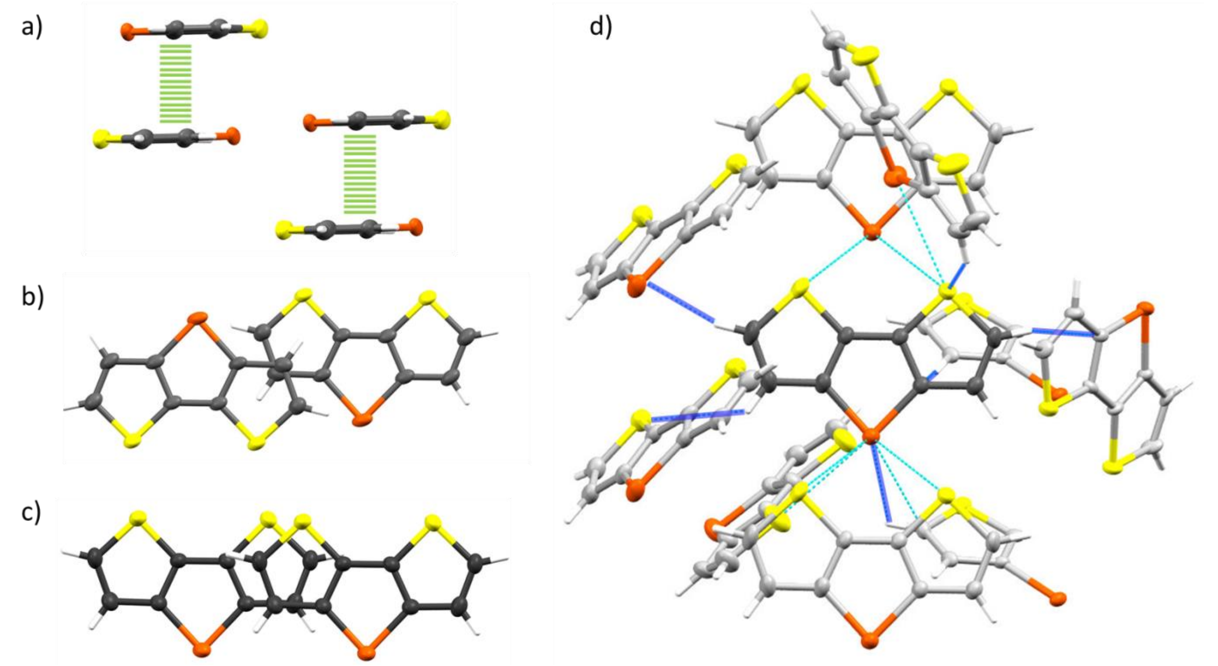


**Figure S6:** Single-crystal X-ray structure analysis of selenolotriacene DTS **2**: (a) partial overlap of two stacked and displaced molecules leading to $\pi$–$\pi$ interactions with distances between 3.27 and 3.40 Å (side view); top view of $\pi$–$\pi$ antiparallel (43% molecular overlap) (b) and parallel (53% molecular overlap) (c) interacting molecules. (d) Intermolecular interactions between heteroatoms and hydrogen-heteroatoms (labelled cyan and blue respectively) for one of the two non-disordered non-equivalent molecules in the reduced unit cell.

**Table S2**: X-ray structure analysis data of heterotriacene DST **3**.

| | | |
|---|---|---|
| Identification code | DST **3** | CCDC 1025419 |
| Empirical formula | $C_8H_4SSe_2$ | |
| Formula weight | 290.09 | |
| Temperature/K | 150.00(10) | |
| Crystal system | monoclinic | |
| Space group | $P2_1/n$ | |
| *a*/Å | 6.02748(19) | |
| *b*/Å | 10.6662(3) | |
| *c*/Å | 12.9279(4) | |
| α/° | 90 | |
| β/° | 96.747(3) | |
| γ/° | 90 | |
| Volume/Å$^3$ | 825.38(4) | |
| Z | 4 | |
| $\rho_{calc}$g/cm$^3$ | 2.334 | |
| μ/mm$^{-1}$ | 9.133 | |
| F(000) | 544.0 | |
| Crystal size/mm$^3$ | 0.237 × 0.142 × 0.114 | |
| Radiation | MoKα (λ = 0.71073) | |
| 2Θ range for data collection/° | 6.346 to 58.688 | |
| Index ranges | -8 ≤ h ≤ 6, -14 ≤ k ≤ 14, -14 ≤ l ≤ 17 | |
| Reflections collected | 6191 | |
| Independent reflections | 2037 [$R_{int}$ = 0.0454, $R_{sigma}$ = 0.0517] | |
| Data/restraints/parameters | 2037/0/101 | |
| Goodness-of-fit on F$^2$ | 1.056 | |
| Final R indexes [I>=2σ (I)] | $R_1$ = 0.0312, w$R_2$ = 0.0568 | |
| Final R indexes [all data] | $R_1$ = 0.0436, w$R_2$ = 0.0632 | |
| Largest diff. peak/hole / e Å$^{-3}$ | 0.61/-0.55 | |

**Table S3**: X-ray structure analysis data of heterotriacene DSS **4**.

| | | |
|---|---|---|
| Identification code | DSS **4** | CCDC 1898450 |
| Empirical formula | $C_{36}H_{18}Se_{13.5}$ | |
| Formula weight | 1516.27 | |
| Temperature/K | 293.15 | |
| Crystal system | monoclinic | |
| Space group | $P2_1/c$ | |
| *a*/Å | 6.108(3) | |
| *b*/Å | 29.049(17) | |
| *c*/Å | 21.949(11) | |
| α/° | 90 | |
| β/° | 91.815(12) | |
| γ/° | 90 | |
| Volume/Å$^3$ | 3892(3) | |
| Z | 4 | |
| $\rho_{calc}$g/cm$^3$ | 2.588 | |
| μ/mm$^{-1}$ | 12.681 | |
| F(000) | 2772.0 | |
| Crystal size/mm$^3$ | 0.221 × 0.085 × 0.044 | |
| Radiation | MoKα (λ = 0.71073) | |
| 2Θ range for data collection/° | 4.598 to 52.778 | |
| Index ranges | -7 ≤ h ≤ 7, -36 ≤ k ≤ 36, -26 ≤ l ≤ 27 | |
| Reflections collected | 74466 | |
| Independent reflections | 7962 [$R_{int}$ = 0.0783, $R_{sigma}$ = 0.0388] | |
| Data/restraints/parameters | 7962/1704/713 | |
| Goodness-of-fit on $F^2$ | 1.233 | |
| Final R indexes [I>=2σ (I)] | $R_1$ = 0.0663, w$R_2$ = 0.1254 | |
| Final R indexes [all data] | $R_1$ = 0.0758, w$R_2$ = 0.1288 | |
| Largest diff. peak/hole / e Å$^{-3}$ | 2.52/-1.63 | |

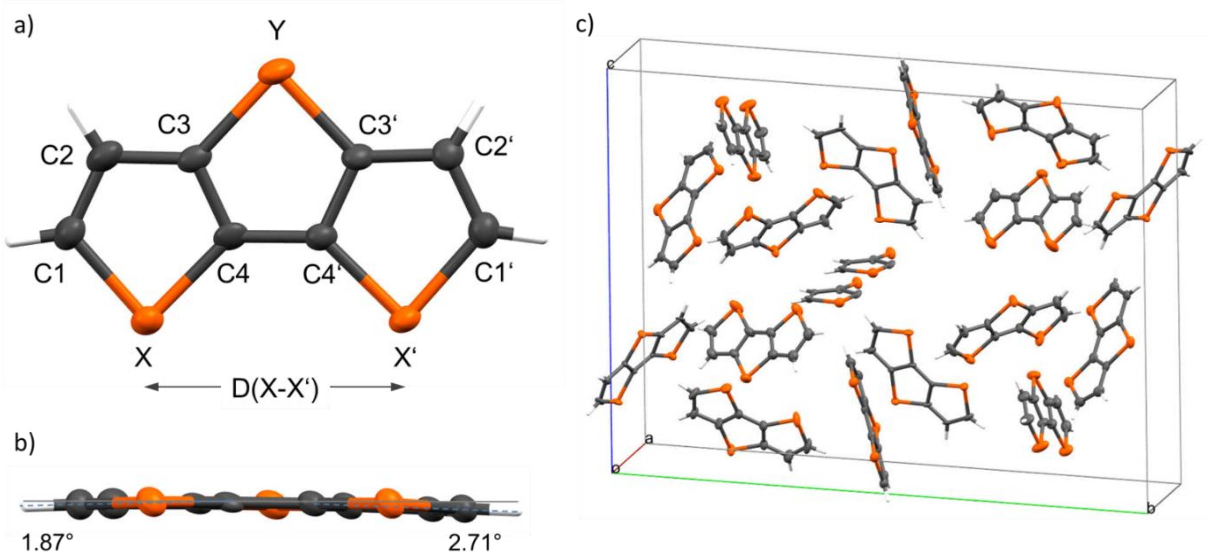


**Figure S7:** Single-crystal X-ray structure analysis of selenolotriacene DSS **4**, (a) individual molecule and atom numbering (top view); (b) side view. (c) Herringbone-type packing structure of the molecules in the unit cell.

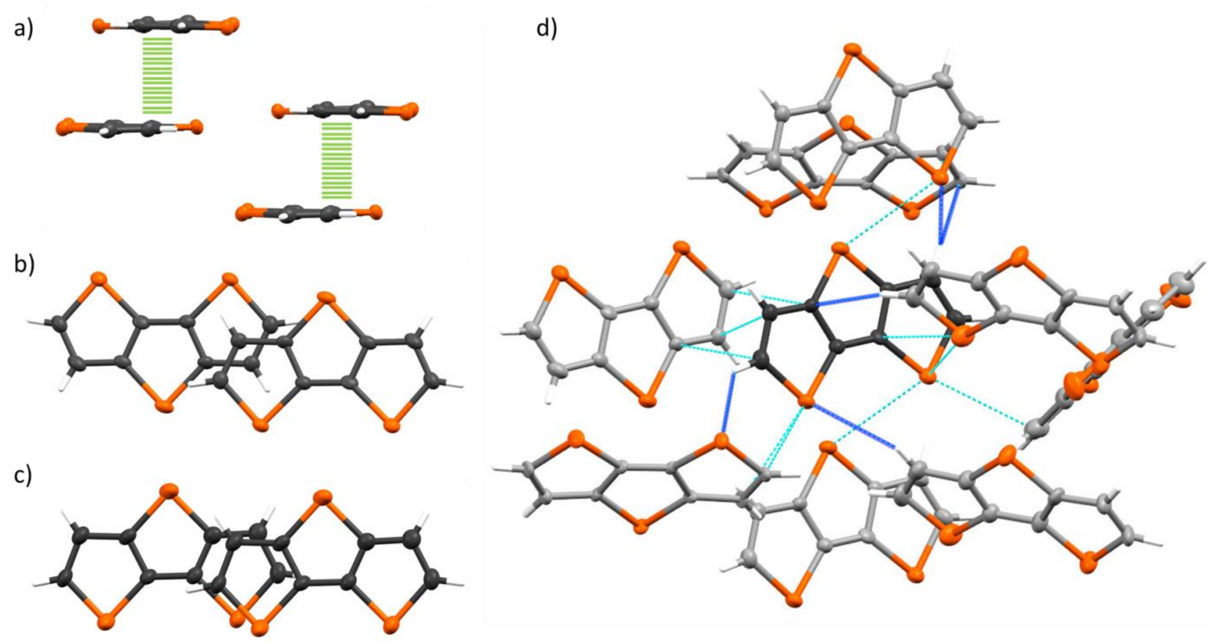


**Figure S8:** Single-crystal X-ray structure analysis of selenolotriacene DSS **4**: (a) partial overlap of two stacked and displaced molecules leading to $\pi$–$\pi$ interactions with distances between 3.34 and 3.38 Å (side view); top view of $\pi$–$\pi$ antiparallel (45% molecular overlap) (b) and parallel (52% molecular overlap) (c) interacting molecules. (d) Intermolecular interactions between heteroatoms and hydrogen-heteroatoms (labelled cyan and blue respectively) for one of the two non-disordered non-equivalent molecules in the reduced unit cell.

**Table S4**: Short contacts for one molecule in the asymmetric unit of DST **3** (a) and DTT **1** (b) from ref. [1,2].

a)

| DST **3** | | | | |
|---|---|---|---|---|
| Atom1 | Atom2 | Distance | Mol. 1 | Mol. 2 |
| Se2 | S3 | 3,645 | x,y,z | -1+x,y,z |
| Se1 | H5 | 3,028 | x,y,z | 1/2-x,-1/2+y,1/2-z |
| H8 | S3 | 2,819 | x,y,z | -1/2+x,1.5-y,1/2+z |

b)

| DTT **1** | | | | |
|---|---|---|---|---|
| Atom1 | Atom2 | Distance | Mol. 1 | Mol. 2 |
| S2 | S1 | 3,566 | x,y,z | 1-x,-1/2+y,1/2-z |
| S2 | S2 | 3,537 | x,y,z | 1-x,-1/2+y,1/2-z |

**Table S5**: Short contacts for one molecule in the asymmetric unit of DTS **2**.

| DTS **2** | | | | |
|---|---|---|---|---|
| Atom1 | Atom2 | Distance | Mol. 1 | Mol. 2 |
| Se1 | S6 | 3,544 | x,y,z | -1+x,y,z |
| Se1 | S8 | 3,605 | x,y,z | -1+x,y,z |
| H28 | S9 | 2,956 | x,y,z | -2+x,y,z |
| H9 | C21 | 2,824 | x,y,z | -1+x,y,z |
| S6 | Se3 | 3,498 | x,y,z | x,y,z |
| S6 | H6 | 2,974 | x,y,z | x,y,z |
| Se1 | S13 | 3,413 | x,y,z | -1+x,1.5-y,-1/2+z |
| H23 | C27 | 2,854 | x,y,z | x,y,z |
| S6 | S11 | 3,547 | x,y,z | 1+x,y,z |
| Se1 | C3AA | 3,348 | x,y,z | -1-x,1-y,-z |
| Se1 | H3AA | 2,92 | x,y,z | -1-x,1-y,-z |
| Se2 | S7 | 3,641 | x,y,z | -1+x,y,z |
| C16 | C16 | 3,267 | x,y,z | 2-x,2-y,-z |
| C16 | C21 | 3,399 | x,y,z | 2-x,2-y,-z |
| H32 | C30 | 2,863 | x,y,z | -x,1/2+y,1/2-z |
| H32 | C33 | 2,794 | x,y,z | -x,1/2+y,1/2-z |

**Table S6**: Short contacts for one molecule in the asymmetric unit of DSS **4**.

| DSS **4** | | | | |
|---|---|---|---|---|
| Atom1 | Atom2 | Distance | Mol. 1 | Mol. 2 |
| Se6 | Se1 | 3,657 | x,y,z | -1+x,y,z |
| C4 | C4 | 3,348 | x,y,z | -x,-y,2-z |
| C8 | C14 | 3,395 | x,y,z | -x,-y,2-z |
| Se9 | H19 | 3,038 | x,y,z | x,1/2-y,1/2+z |
| Se6 | Se8 | 3,765 | x,y,z | 1+x,1/2-y,1/2+z |
| C8 | H9 | 2,8 | x,y,z | 1+x,1/2-y,1/2+z |
| C13 | Se8 | 3,578 | x,y,z | 1+x,1/2-y,1/2+z |
| H0AA | C17 | 2,857 | x,y,z | -x,-1/2+y,1.5-z |
| Se9 | C22 | 3,457 | x,y,z | x,1/2-y,1/2+z |
| H14 | Se5 | 3,092 | x,y,z | x,1/2-y,1/2+z |
| Se6 | C16 | 3,569 | x,y,z | x,y,z |
| Se4 | Se2 | 3,65 | x,y,z | -1+x,y,z |
| Se8 | Se2 | 3,667 | x,y,z | -1+x,y,z |
| Se2 | Se1A | 3,52 | x,y,z | x,y,z |
| H19 | Se1A | 3,064 | x,y,z | x,y,z |
| Se4 | Se11 | 3,546 | x,y,z | -1+x,1/2-y,-1/2+z |
| Se4 | H1 | 3,017 | x,y,z | -1+x,1/2-y,-1/2+z |
| C2 | H1AA | 2,88 | x,y,z | -1+x,y,z |
| Se2 | C3AA | 3,417 | x,y,z | x,y,z |
| Se2 | H3AA | 3,03 | x,y,z | x,y,z |
| Se10 | Se2A | 3,564 | x,y,z | x,y,z |
| Se1A | H3AA | 3,089 | x,y,z | x,y,z |

## XRD measurements

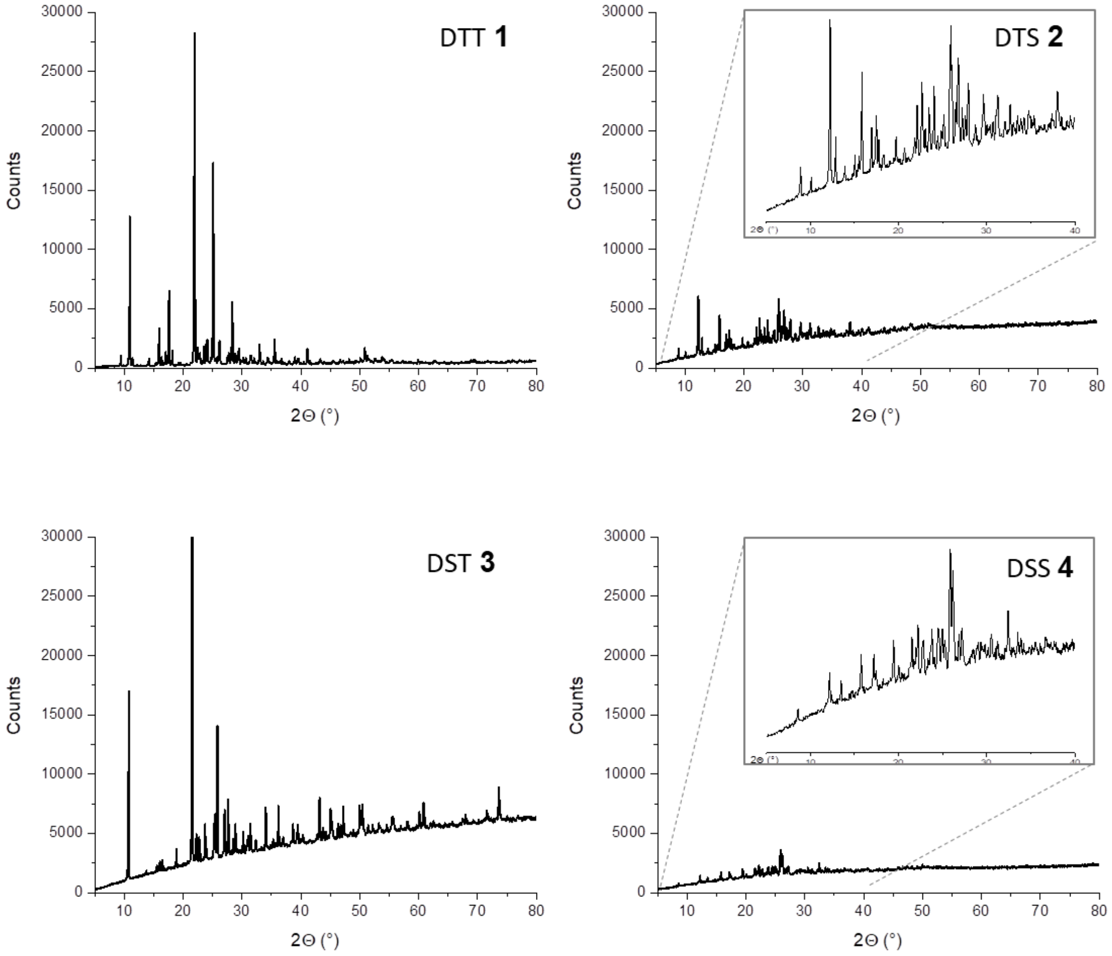


**Figure S9:** XRD plots of heterotriacenes **1**–**4**. Insets: magnification of the region 2Θ = 5–40°.

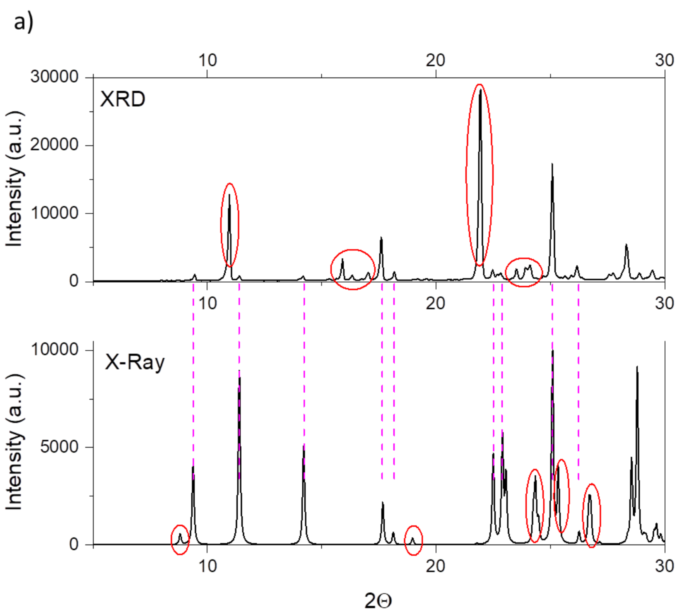


b)

| | X-Ray | | XRD |
|---|---|---|---|
| | Miller Indices | $d_{hlk}$ [Å] (Peak Intensity) | $d_{hlk}$ [Å] (Peak Intensity) |
| | | 10.02 (5.7%) | — |
| | 002 | 9.42 (42.1%) | 9.36 (3.8%) |
| | | | 8.07 (45.1%) |
| | 10-2 | 7.76 (89.7%) | 7.77 (2.9%) |
| | 10-2 | 6.23 (50.9%) | 6.24 (2.8%) |
| | — | | 5.57 (11.9%) |
| | 200 | 5.02 (21.8%) | 5.04 (23.1%) |
| | 20-2 103 | 4.89 (6.4%) | 4.87 (5.2%) |
| | 10-4 | 4.67 (3.4%) | — |
| | — | | 4.05 (100%) |
| | 104 | 3.95 (47.1%) | 3.95 (6.1%) |
| | 20-4<br>011 | 3.88 (58.6%)<br>3.86 (38.7%) | 3.89 (4.4%)<br>— |
| | 11-1 | 3.65 (35.4%) | — 3.68 (8.4%) |
| | 111 | 3.54 (100%) | 3.55 (61.2%) |
| | 11-2 | 3.51 (41.2%) | — |
| | 30-2 | 3.39 (7.0%) | 3.40 (8.2%) |

**Figure S10:** XRD plot and X-Ray powder pattern of DTT **1** (a), including missing reflexions (red encircled). Table with $d_{hkl}$ and relative intensity of X-Ray and XRD reflexions for selected Miller indices.

a)

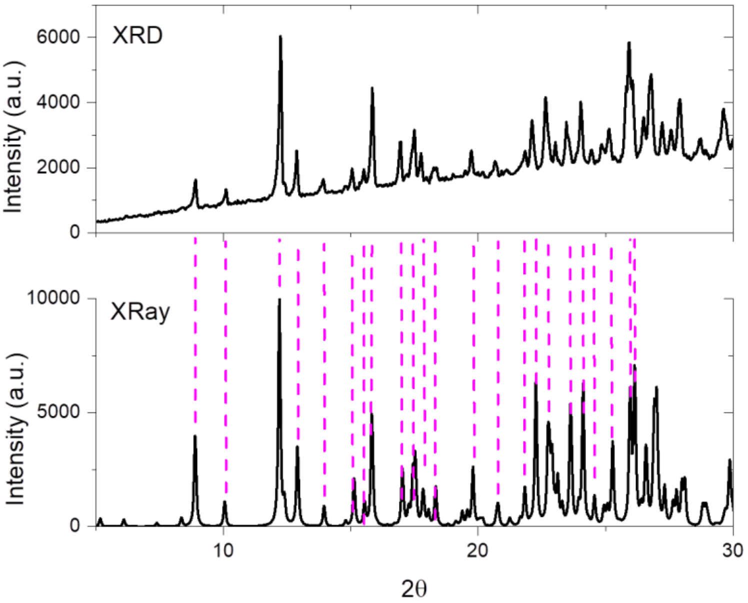


b)

| X-Ray | | XRD |
|---|---|---|
| Miller indices | $d_{hlk}$ [Å] (Peak Intensity) | $d_{hlk}$ [Å] (Peak Intensity) |
| <0 1 2> | 9.93 (40%) | 9.92 (18%) |
| <0 3 1> | 8.80 (11%) | 8.75 (10%) |
| <0 4 0> | 7.23 (100%) | 7.22 (100%) |
| <0 1 3> | 6.48 (35%) | 6.87 (29%) |
| <1 1 0> | 5.83 (22%) | 5.88 (13%) |
| <0 3 3> | 5.70 (11%) | 5.71 (12%) |
| <1 1 1> | 5.59 (50%) | 5.59 (61%) |
| <0 1 4> | 5.20 (26%) | 5.22 (26%) |
| <0 4 3> | 5.05 (33%) | 5.07 (32%) |
| <1 2 -2> | 4.96 (16%) | 4.99 (18%) |
| <0 6 0> | 4.82 (18%) | 4.83 (8%) |
| <0 5 3> | 4.47 (27%) | 4.50 (16%) |
| <0 4 4> | 4.27 (11%) | 4.30 (9%) |
| <1 3 3> | 4.07 (18%) | 4.02 (31%) |
| <0 6 3> | 3.99 (64%) | 3.92 (44%) |
| <1 5 -2> | 3.90 (46%) | 3.79 (28%) |
| <1 6 0> | 3.76 (54%) | 3.70 (39%) |
| <1 6 1> | 3.69 (63%) | 3.64 (9%) |
| <1 3 4> | 3.61 (14%) | 3.58 (12%) |
| <1 6 2> | 3.51 (38%) | 3.54 (21%) |
| <0 2 6> | 3.43 (61%) | 3.43 (72%) |
| <1 0 5> | 3.40 (70%) | 3.36 (26%) |
| <1 6 -3> | 3.35 (36%) | 3.23 (51%) |
| <1 3 5> | 3.30 (61%) | |

**Figure S11:** XRD plot and X-Ray powder pattern of DTS **2** (a). Table with $d_{hkl}$ and relative intensity of X-ray and XRD reflexions for selected Miller indices.

a)

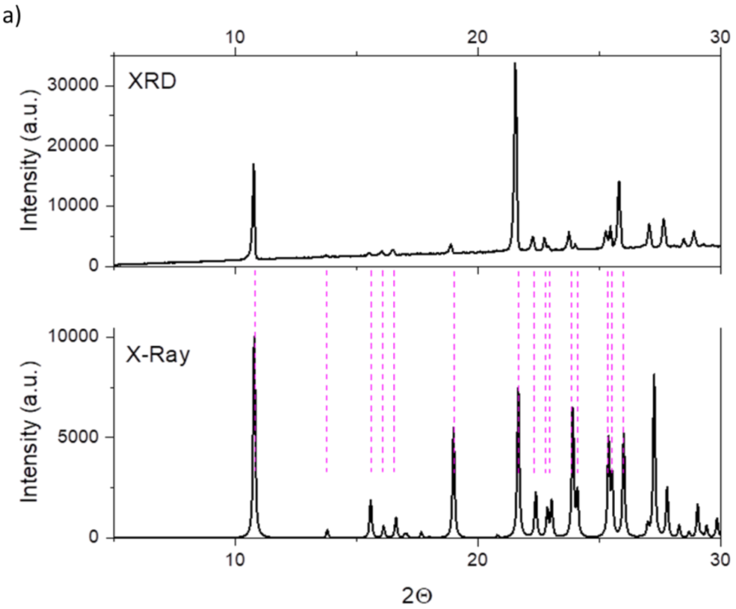


b)

| X-Ray | | XRD |
|---|---|---|
| Miller indices | $d_{hlk}$ [Å] (Peak Intensity) | $d_{hlk}$ [Å] (Peak Intensity) |
| <0 1 1> | 8.20 (100%) | 8.22 (51.2%) |
| <0 0 2> | 6.42 (4.1%) | 6.45 (1%) |
| <1 0 -1> | 5.69 (19.0%) | 5.70 (1.4%) |
| <0 1 2> | 5.50 (6.2%) | 5.52 (2.5%) |
| <0 2 0> | 5.34 (10.2%) | 5.37 (3.1%) |
| <1 0 -2> | 4.67 (54.8%) | 4.70 (4.3%) |
| <0 2 2> | 4.10 (74.9%) | 4.13 (100%) |
| <0 1 3> | 3.97 (23.0%) | 3.96 (7.4%) |
| <1 2 -2> | 3.89 (15.5%) | 3.91 (6.2%) |
| <1 1 -3> | 3.86 (19.2%) | 3.89 (10%) |
| <1 2 1> | 3.72 (65.1%) | 3.75 (2.7%) |
| <1 2 -2> | 3.51 (50.8%) | 3.53 (11.1%) |
| <0 3 1> | 3.43 (52.3%) | 3.45 (47.5%) |
| <1 2 2> | 3.27 (81.5%) | 3.30 (12%) |

**Figure S12:** XRD plot and X-ray powder pattern of DST **3** (a). Table with $d_{hkl}$ and relative intensity of X-ray and XRD reflexions for selected Miller indices.

## Electrochemical characterization of heterotriacenes 1–4

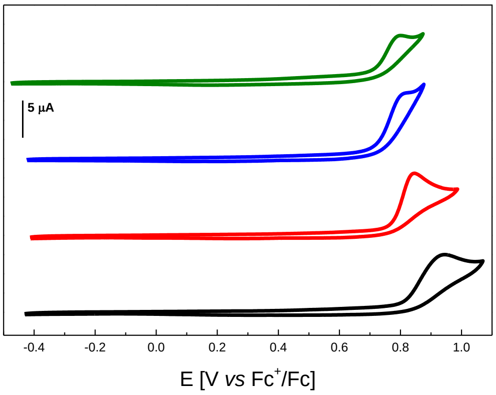


**Figure S13:** Cyclic voltammograms (acetonitrile / $TBAPF_6$ ($10^{-3}$ M) scan rate = 100 mV $s^{-1}$) of DTT **1** (in black), DTS **2** (in red), DST **3** (in blue), and DSS **4** (in green).

## Electrochemical polymerization of heterotriacenes DTT 1, DST 3, and DSS 4 and characterization of corresponding polymers P1, P3, and P4

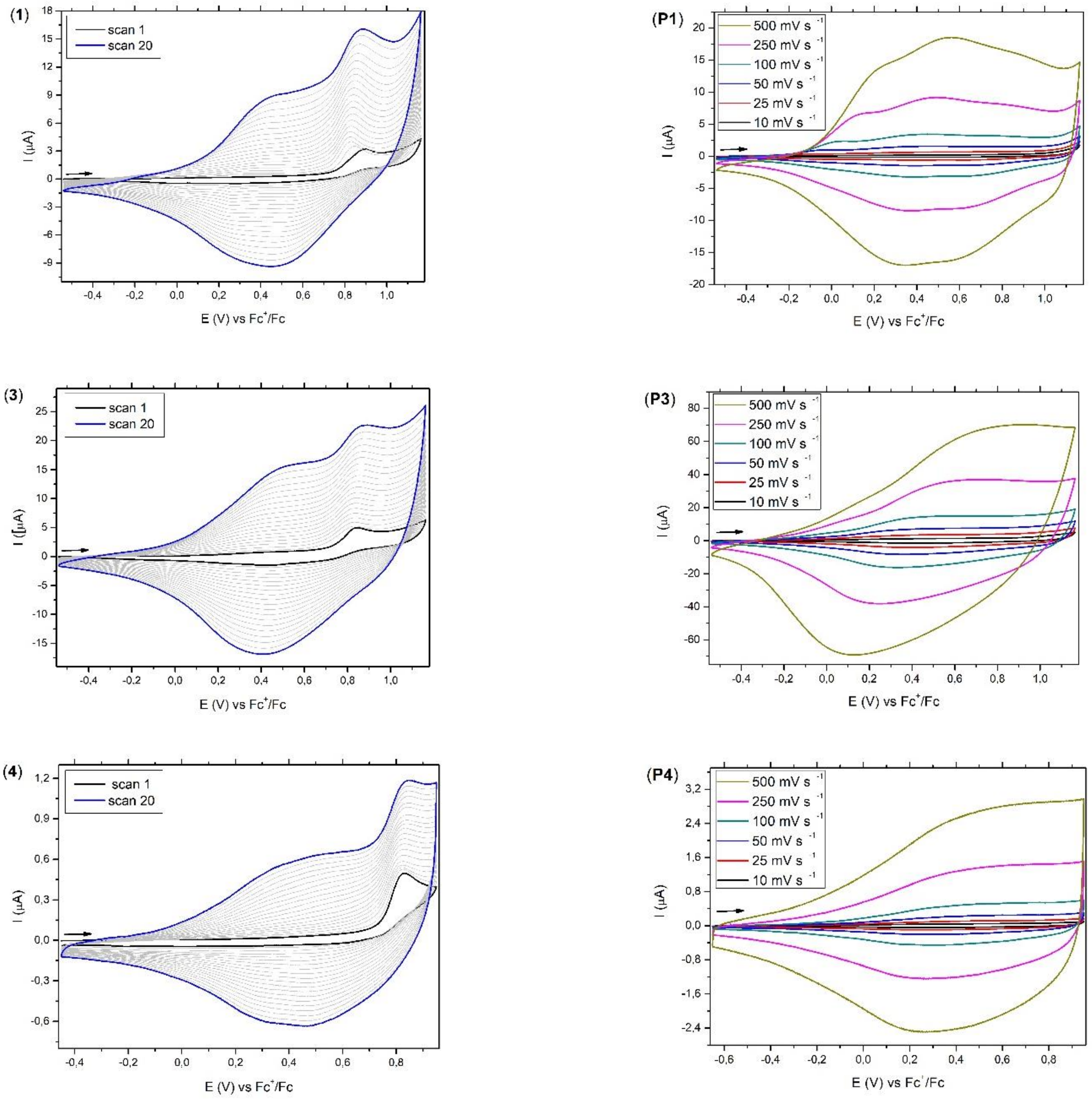


**Figure S14:** Multisweep voltammograms for the electrochemical polymerization of monomeric heterotriacenes DTT **1**, DST **3**, and DSS **4** in DCM/$TBAPF_6$ (0.1 M) at a scan rate of 100 mV $s^{-1}$ (left) and electrochemical characterization of the corresponding polymers **P1**, **P3**, and **P4** in monomer-free electrolyte DCM/$TBAPF_6$ (0.1 M) at different scan rates (10–500 mV $s^{-1}$) (right).

## Multisweep voltammograms and cycling stability for poly(heterotriacenes) P1–P4

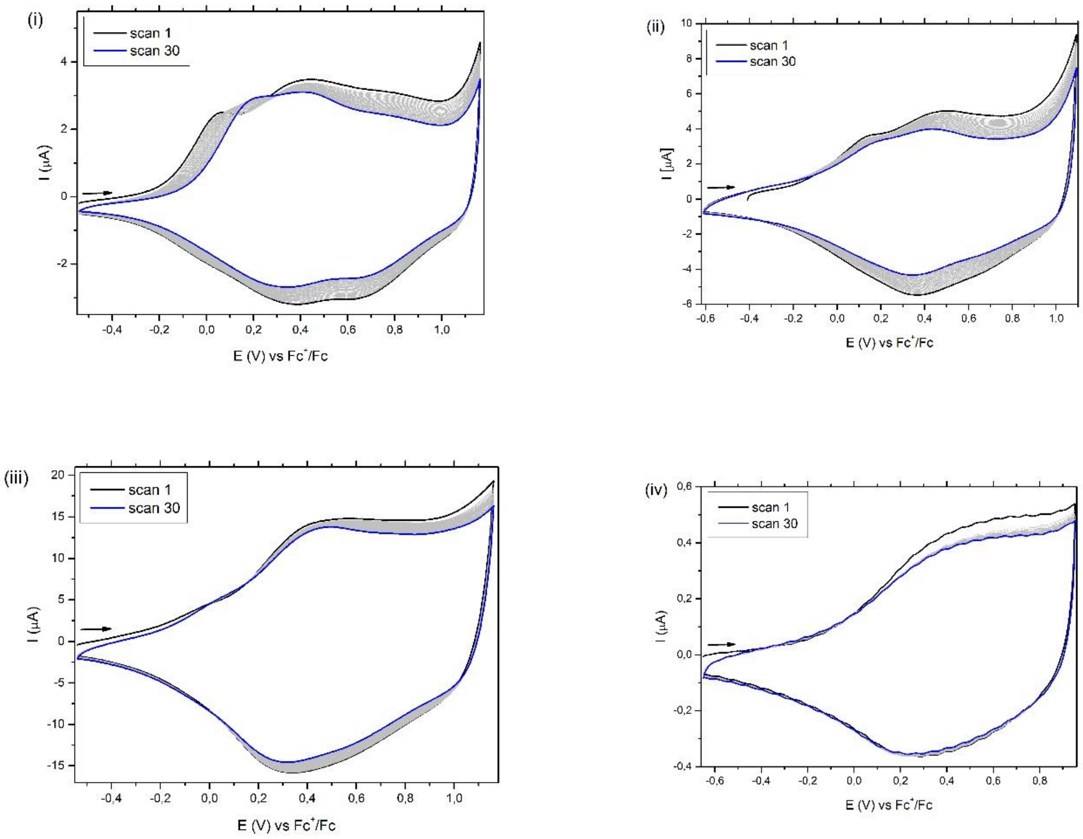


**Figure S15:** Multisweep voltammogram (30 scans) for the electrochemical cycling stability of polymer **P1** (i), **P2** (ii), **P3** (iii), and **P4** (iv) in DCM/$TBAPF_6$ (0.1 M) at a scan rate of 100 mV $s^{-1}$.

## Spectroelectrochemistry of poly(heterotriacenes) P1–P4

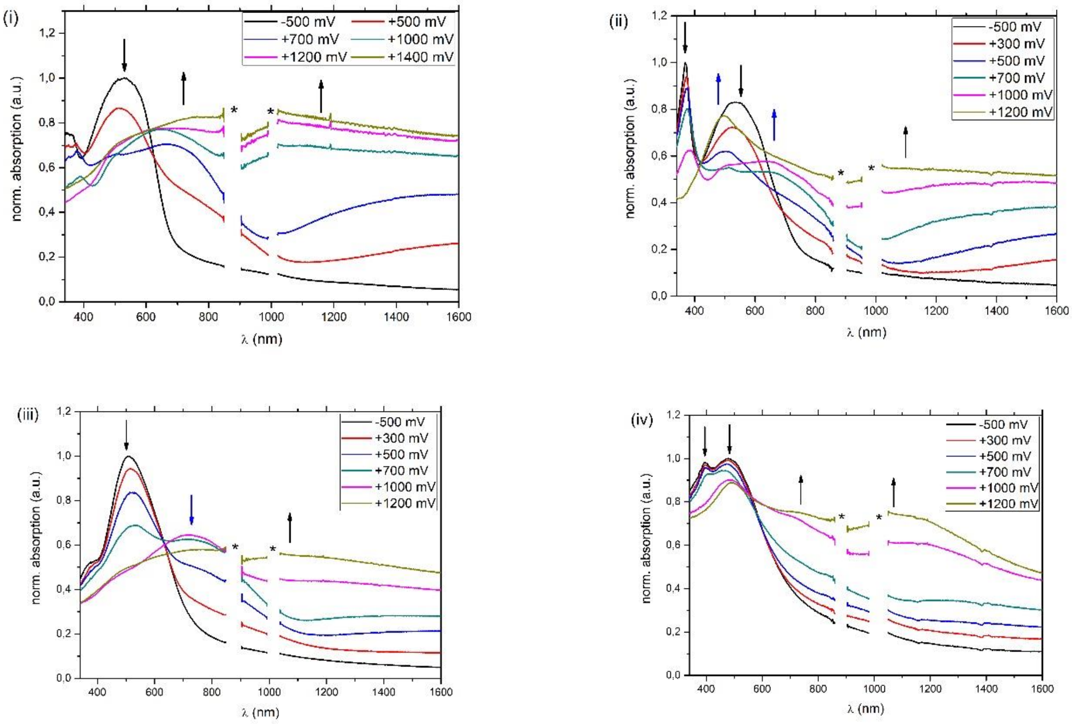


**Figure S16:** UV–vis–NIR spectra obtained from spectroelectrochemical measurements of polymer **P1** (i), **P2** (ii), **P3** (iii), and **P4** (iv). Applied voltages are stated vs. Ag/AgCl. Artefacts are marked with * in the spectra. Black arrows show the changes starting at low potentials, blue arrows show the further changes at high potentials.

**Table S1**: Optical properties of poly(heterotriacenes) **P1**–**P4**. The values of $\lambda_{max}$, $\lambda_{onset}$, and the energy gap $E_g$ were obtained from UV–vis–NIR spectra of neutral polymer films measured in a spectroelectrochemical setup. The maxima $\lambda_{max, ox}$ of the oxidized films were obtained from spectroelectrochemical measurements. The LUMO energy levels were calculated from the HOMO energy level (Table 3) and $E_g$, respectively.

| Polymer | $\lambda_{max}$ [nm] | $\lambda_{onset}$ [nm] | $E_g$ [eV] | LUMO [eV] | $\lambda_{max, ox}$ [eV] |
|---|---|---|---|---|---|
| **P1** (PDTT) | 532 | 692 | 1.79 | -3.13 | 763 (br) |
| **P2** (PDTS) | 532 (369) | 744 | 1.67 | -3.28 | 1090 (497, 629 (sh)) |
| **P3** (PDST) | 509 | 748 | 1.66 | -3.27 | 773 (br) |
| **P4** (PDSS) | 478 (395) | 744 | 1.67 | -3.31 | 1090 (493, 703 (sh)) |
| **PDTP** (S NH S) [3] | 524 | 665 | 1.86 | -2.70 | 1079 (br) |

## References

1. Bertinelli, F.; Palmieri, P.; Stremmenos, C.; Pelizzi, G.; Taliani, C. *J. Phys. Chem.* **1983,** *87,* 2317–2322.
2. Castañeda, R.; Khrustalev, V. N.; Fonari, A.; Brédas, J.-L.; Getmanenko, Y. A.; Timofeeva, T. V. *J. Mol. Struct.* **2015,** *1100,* 506–512.
3. Förtsch, S.; Bäuerle, P. *Polym. Chem.*, **2017**, *8*, 3586-3595.
